# Migrant penalty in the European labor markets: the interplay between individual characteristics and the regional context

**DOI:** 10.3389/fsoc.2023.1270167

**Published:** 2023-11-01

**Authors:** Maurizio Avola, Giorgio Piccitto, Federico Vegetti

**Affiliations:** ^1^Department of Political and Social Sciences, University of Catania, Catania, Italy; ^2^Department of Political and Social Sciences, University of Bologna, Bologna, Italy; ^3^Department of Cultures, Politics and Society, University of Turin, Turin, Italy

**Keywords:** migrant penalty, regional labor market, education, employment, job quality, Europe

## Abstract

This study aims to verify if and how migrant penalty in the labor market is associated with sub-national characteristics, exploring the relevance of the regional occupational structure. We expect that a greater relevance of the share of low-status jobs at the regional level reduces the migrant penalty in terms of the probability of being employed, but increases the gap with natives in terms of job quality. We investigate this *trade-off* by estimating a set of hierarchical models on the EU-LFS data (2009–2015) for 19 countries and 189 regions. Results suggest a pattern consistent with the trade-off hypothesis, nuanced by heterogeneity at the individual level: in regions where the share of low-status jobs is higher, mid-high educated immigrants from less developed countries are less (or not) penalized compared to natives in terms of employment, while they face a stronger penalty in terms of job quality. What is more, the trade-off is not observed when considering low-educated migrants or those from high-income countries.

## Introduction

Immigration is arguably the socio-economic phenomenon that more than any other is changing European societies in recent years, making them more and more multi-ethnic. Geographical origin has become an increasingly relevant factor of stratification like gender, social origin and education, as migrants represent nowadays a structural component of the demand and supply in European labor markets. In this context, many scholars have compared the labor market performance of immigrants and natives to shed light on possible different mechanisms of socio-economic integration. This literature has found a so-called *migrant* (or *ethnic*) *penalty*, defined from a micro-level perspective as the remaining difference in immigrants' and natives' labor market achievement when socio-demographic characteristics are controlled for (Berthoud, 2000; Carmichael and Woods, 2000; Heath and Cheung, 2007).

Several studies on Europe highlighted the existence of a migrant penalty in terms of both probability of being employed (or avoiding the risk of unemployment) and of having a high-skilled job (Kalter and Kogan, 2006; Kogan, 2006, 2007; Fleischmann and Dronkers, 2007, 2010; Heath and Cheung, 2007; Pichler, 2011; Reyneri and Fullin, 2011a,b; Ballarino and Panichella, 2015, 2018; Gorodzeisky and Semyonov, 2017). These studies have emphasized how the migrant penalty is moderated by the macro-institutional context, considering the role of some key features at the national or supra-national level (welfare regime, model of capitalism, labor market regulation, migration or immigrants' integration policies, occupational structure). Particularly, these studies highlight a distinction between the *double penalty* model and the *trade-off* model of migrants' labor market integration (Panichella, 2018): in Central-Northern Europe, migrants face a double penalty with respect to natives in terms of employment and job quality; in Southern Europe, natives and migrants have similar chances to be employed, but the latter are strongly penalized in terms of getting a high-skilled job.

This macro-level perspective overlooks the fact that, within the same country, different patterns of immigrants' labor market insertion and penalty at the local or meso-level may coexist (Avola, 2015; Cantalini et al., 2022). Since different contexts within the same country share the same formal institutional setting, the within-country heterogeneity in patterns of immigrants' occupational integration should be related to the structure and the informal regulation of the labor markets. In particular, we refer to the relevance of the secondary labor market (Kogan, 2006; Fleischmann and Dronkers, 2010; Reyneri and Fullin, 2011a) and the role of informal and inherently *locally-rooted* institutions, like social norms and practices, collective beliefs and conventions (Tödtling and Trippl, 2005; Iammarino et al., 2019), which connote in particular the area of peripheral jobs. For this reason, while recognizing the importance of the national (or supra-national) dimension, to improve the accuracy of the analysis we use the regional level as the finest-grained one available to estimate the moderating effect of the labor market structure on the model and extent of the migrant penalty in Europe.

We use seven waves (2009–2015) of the European Labor Force Survey (EU-LFS) data to explore the migrant penalty from a meso-level perspective, focusing on 189 regions in 19 countries, considering both the probability of being employed and the job quality. Moreover, we look at the moderating role of gender and education, as crucial factors that determine the different performance of natives and migrants in the labor market.

Our findings reveal several interesting patterns. First, we confirm the presence of a pattern consistent with the trade-off model of migrant labor market integration: when the share of low-status jobs in a region is higher, migrants from less developed countries are more likely than natives to find a job, but more often of low-quality. Second, we find a moderating role of education: while the trade-off is stronger for middle or highly educated individuals, it doesn't occur for people with low education.

## The migrant penalty in Europe: literature overview, research questions and hypothesis

### Micro-level perspective

The literature on the migrant penalty in labor markets has looked at phenomena such as professional status, on the one hand, and job quality, on the other.

This literature has mainly adopted a micro-level approach, studying the association between these outcomes and individual characteristics. Following the human capital perspective, many studies suggest that the gap between migrants and natives arises because immigrants have to adapt their credentials and skills to the local labor demand: in fact, education is mainly a country-specific asset, not easily transferable in a different socio-economic context (Borjas, 1994; Friedberg, 2000; Chiswick and Miller, 2009; Dustmann and Glitz, 2011). Foreign qualifications, for example, may lack formal recognition in the host country institutions and have a weak informal signaling function, hampering the labor market achievement.

But there are also other individual factors driving the migrant penalty: with respect to natives, migrants are likely to have a limited host language proficiency (Dustmann and Glitz, 2011; Chiswick and Miller, 2015; Koopmans, 2016), different “work cultures” and attitudes (Friberg, 2012), a lower knowledge of the host labor market functioning (Chiswick, 1978; Kogan, 2007), and a mainly bonding, instead of bridging, social capital (Lancee, 2012; Koopmans, 2016; Avola and Piccitto, 2020; Leschke and Weiss, 2020).

Additionally, the perceived temporariness of their stay in the host country leads to different labor market strategies and makes them less willing to invest in country-specific endowments and resources (Piore, 1979; Dustmann, 2000). For this reason, Kalter and Kogan (2006) argue that the migrant penalty in the labor market is also the result of a self-exclusion process of immigrants that can trigger a vicious circle for which migrants remain trapped in the secondary labor market (Grubanov-Boskovic and Natale, 2017), characterized by low-status, low-paid and precarious job positions (Kogan, 2004; Reyneri and Fullin, 2011a) and low opportunities of upward social mobility, especially in more segmented labor markets (Simón et al., 2014; Fernández-Macías et al., 2015; Fellini and Guetto, 2019; Avola and Piccitto, 2020; Panichella et al., 2021).

Finally, the migrant penalty may also be the result of direct or indirect discriminatory practices exerted by the institutions, like legal restrictions prohibiting foreigners from accessing some occupations, and employers. The latter can be motivated by hostility toward certain ethnic groups, particularly if they are perceived as visibly different from the native population, for cultural, religious or phenotypical reasons (taste-based discrimination), or if migrants belong to an ethnic group labeled negatively because considered less serious, reliable and productive (statistical discrimination) (Becker, 1971; Arrow, 1972; Zschirnt and Ruedin, 2016; Koopmans et al., 2019; Di Stasio and Lancee, 2020).

### Macro-level perspective

The extent and the structure of the migrant penalty are also moderated by different macro-institutional characteristics of the host society. However, the studies adopting this macro-level perspective in the European context have not always been consistent in their results. The first pioneering research of Kogan (2006, 2007) shows that migrant penalty, in terms of risk of unemployment and ISEI score, is lower in liberal welfare states (Ireland and UK), characterized by higher freedom of hiring and firing and lower unemployment benefits, if compared with conservative (Central and Southern European countries) and social democratic regimes (Scandinavian countries). Differently, Fleischmann and Dronkers (2010) do not find any significant association between welfare regimes and the chance of avoiding unemployment for migrants, while Pichler (2011) shows that only in the Mediterranean welfare regime (Cyprus, Greece, Italy, Portugal and Spain) immigrants are disadvantaged in the chance of achieving a “good job” (EGP I-II).

Another macro-institutional feature often considered in the research on migrant penalty is the regulation of the labor market: considering that hiring an immigrant could be more risky for employers (for the reasons discussed in the previous paragraph), a growing migrant penalty could be expected as the rigidity of labor regulation increases. Also in this case, results are ambiguous. Whereas, Kogan (2006) finds a significant negative effect of the EPL (Employment Protection Legislation index) on unemployment for male migrants, the same outcome is not significant in the studies by Fleischmann and Dronkers (2010) and Bisin et al. (2011). At the same time, Reyneri and Fullin (2011a) show a lower unemployment risk for non-EU migrants in those countries where the EPL is higher,^1^ confirming a previous hypothesis tested by Angrist and Kugler (2003) and Sá (2008). On the other hand, when considering job quality as an outcome, a greater rigidity of labor regulation seems in some cases to affect the chance of achieving a high occupational status for immigrants more than for natives (Fleischmann and Dronkers, 2007), but not in others (Pichler, 2011).

Unexpectedly, the Migration Integration Policy Index (MIPEX) or other indicators of immigration policy seem to be not relevant in terms of migrant penalty reduction for employment or unemployment, class attainment and income (Büchel and Frick, 2005; Fleischmann and Dronkers, 2010; Bisin et al., 2011; Pichler, 2011; Kogan, 2016).

Differently from what emerged when considering welfare regimes and labor market regulation, the association between the labor market structure and the migrant penalty is more robust. Indeed, from several studies it emerges that the migrant penalty in terms of unemployment risk is lower when the share of low-status occupations is higher (Kogan, 2006; Fleischmann and Dronkers, 2010; Reyneri and Fullin, 2011a): as suggested by the segmented labor market theory (Piore, 1979; Massey et al., 1993; Reich, 2008), the “poor” jobs at the bottom of the occupational hierarchy, with poor social recognition and low chances of upward mobility, are often avoided by the “core” native labor force and filled by the “peripheral” migrant workforce.

Moreover, also employers can contribute to this “ethnicization” of the labor market. As suggested by Auer et al. (2019, p. 97), ≪when assessing their candidates, employers take both the social distance perception and the occupational hierarchy into account≫. This means that if natives or immigrants perceived as socio-culturally closer are preferred when hiring processes concern jobs at the top occupational hierarchy, ≪whenever an occupation conveys the image of being “unsuitable” or “unattractive” for a native worker, an immigrant background almost automatically signals a better fit for employers≫ (Auer et al., 2019). Last, as Kogan (2007) and Reyneri and Fullin (2011a) highlight, if protection against firing procedures can influence employers' hiring of immigrants in the primary labor market, this is less relevant in the secondary one, where formal labor regulation can be bypassed by informal rules.

Other particularly informative results on the relevance of macro-institutional context in the structure and the extent of migrant penalty in Europe come from cross-country analyses using measures of both professional status and job quality. First of all, a research project focusing on the migrant penalty in six countries shows that in Italy and Spain the probability of being employed (or of avoiding the risk of unemployment) is not very different between immigrants and natives, but the former are much more penalized in terms of access to high-skilled occupations; at the same time, in Denmark and the Netherlands immigrants suffer a greater risk of unemployment but a relatively small penalty in terms of the quality of the job, while Germany and the UK are in an intermediate position (Zorlu and Hartog, 2008; Demireva, 2011; Reyneri and Fullin, 2011b). These findings are almost entirely confirmed by studies considering separately men (Ballarino and Panichella, 2015) and women (Ballarino and Panichella, 2018) or expanding the number of countries considered (Panichella, 2018). To sum up, in Central-Northern European countries the migrant penalty concerns both the probability of being employed (or avoiding unemployment) and the access to high-skilled jobs (double penalty model), while in Southern Europe, characterized by less qualified and more segmented labor markets, natives and immigrants have similar chances of being employed but remarkably diverging probabilities of getting a good job (trade-off model).

### The advantages of a regional approach

Even though the national/macro-institutional context contributes to shaping the migrant penalty, sub-national analyses (Avola, 2015; Cantalini et al., 2022) show that, within the same country, considerably different patterns of immigrant labor market integration coexist. These within-country differences cannot be attributed to institutional characteristics: factors like welfare regime, model of capitalism, formal regulation of the labor market and migration policies are indeed shared by all the territorial sub-units within the same country. Hence, such a meso-level heterogeneity should be referred to the structure and mechanisms of informal regulation of labor markets, which largely differ within a given institutional and socio-economic context. Of great concern, in this sense, is the relevance of the secondary labor market, as the locus where informal practices of labor regulation and demand-supply matching are at most at play (Kogan, 2006; Fleischmann and Dronkers, 2010; Reyneri and Fullin, 2011a).^2^ These features, integrated with those referring to the individual socio-demographic characteristics, are particularly important for the advancement of the understanding of migrants' achievements in the host society (Gertler, 2003; Boschma, 2017). Indeed, globalization and processes of outsourcing have determined that economic competition is less and less country-localized and enrooted within national countries; conversely, these two dynamics have made extremely crucial the regional dimension, up to the point that region can be seen as ≪an essential level of economic coordination in capitalism≫ (Storper, 1995, p. 192). This mechanism is described by the “location paradox” (Porter, 2000) for which the more things are mobile, the more decisive location becomes: the most enduring competitive advantage in a global economy is indeed local. Firms develop their competitiveness in interaction with local capabilities, which have been defined as based on “the region's infrastructure and built environment; the natural resources accessible in the region; the region's specific institutional endowment; the knowledge and skills available in the region” (Maskell and Malmberg, 1999, p. 173). Hence, spatial proximity is a key factor in the transmission of tacit endowments and, as such, in shaping labor market transactions and behaviors.

Following these insights on the role of the host labor market structure in moderating the migrant penalty, a regional perspective can have manifold advantages. First, this perspective accounts for the growing within-country heterogeneity at the socio-economic and occupational level observed in Europe (Hurley et al., 2019; Rosés and Wolf, 2021; Viesti, 2021). In other words, it provides a more detailed outlook of the labor market structure in terms of the distribution of employment by occupation, economic activity, professional status, etc. Second, many informal institutions like social norms and practices, collective beliefs and conventions, connoting in particular the matching between demand and supply for peripheral jobs, are inherently *locally-rooted* (Tödtling and Trippl, 2005; Iammarino et al., 2019). A regional approach accounts for the “tacit dimension” of knowledge (Howells, 2002; Polanyi, 2009), which is shared among people having a common social context (shared values, language, and culture) and which has been recognized as one meaningful factor driving the economic behavior (Hodgson, 1988).

### Research question and hypotheses

Our goal in this paper is to investigate the association between migrant penalty and labor market structure, using the regional level as the finest-grained one available to define the latter. As segmented labor market theory suggests, natives are less willing than migrants to offer themselves for poorly paid, unstable, low-skilled and with poor social recognition jobs, and employers tend to prefer immigrants because they better fit with these kinds of low-status jobs. Moreover, in secondary labor markets, a rigorous selection of workers is less important also because employers can more easily dispose of hired workers, profiting by more extensive sources of formal and informal flexibility. Hence, we expect that a higher relevance of the secondary labor market reduces the gap for immigrants in terms of probability of being employed, but that increases the gap in terms of job quality, following a trade-off pattern.

Moreover, we expect individual characteristics to interact with the structure of the labor market, moderating the trade-off effect. First, if migrants who are more socio-culturally distant from the natives face more difficulties in performing well in the host labor market, we should expect the trade-off to be sharper for migrants coming from less developed countries (*hypothesis 1*). Conversely, if immigrants coming from high-income countries are more similar to the natives, they will be less available to offer themselves in the secondary labor market, so a trade-off won't be observed for them (*hypothesis 2*). Second, we look at the moderating role of education. Coherently with the human capital perspective reported above, we expect that the migrant penalty will grow as the level of education increases: if educational qualifications are difficult to be transferred in the host society, and especially in those with a higher prevalence of secondary labor markets, we should expect the trade-off pattern between the probability of being employed and the probability of being segregated in low-quality jobs to be higher for immigrants with higher levels of education (*hypothesis 3*).

## Data, variables and empirical strategy

### Data

We use multilevel modeling to shed light on how the qualification of the labor market at the regional level moderates the probability of being employed and the quality of job by migration status. We use data from the European Labor Force Surveys (EU-LFS) from 2009 to 2015. To provide an overview as comprehensive as possible of the European labor markets, we include in our analysis 189 regions in 19 countries: Austria, Belgium, Czech Republic, Denmark, Germany, Finland, France, Greece, Hungary, Ireland, Italy, Norway, Portugal, Romania, Slovakia, Sweden, Spain, Switzerland, and the United Kingdom.^3^ We focus on NUTS2 regions, as they provide the best balance between geographic detail and data availability/sample representativeness.^4^ Since we are interested in occupational outcomes in the core workforce, we concentrate our analysis on people aged between 25 and 64. People in the armed forces are excluded from the analysis.

### Dependent variables

In order to verify our hypotheses, we define two dependent variables: being employed (1 = employed; 0 = unemployed or inactive) and job quality, proxied by the International Socio-Economic Index of Occupational Status (ISEI) (Ganzeboom and Treiman, 1996).

### Independent variables

Our main independent variable is the respondents' migration status. We combine information on country of birth and nationality to better distinguish different groups of migrants, establishing a progression of socio-economic “closeness” (Auer et al., 2019) between the individuals and the country where they live. First of all, we define as “natives” those who were born and who have the nationality of the country where they live, and as “migrants” those who were born and who have the nationality of another country. In order to isolate first generations, given how relevant the acquisition of citizenship can be for the labor market performance of immigrants (Catron, 2019), we include those who were born in the country where they live and have a different nationality (mainly second-generation not naturalized) and those who were born in a different country and have the nationality of the country where they live (naturalized immigrants) in a residual category (*mixed-status*). Moreover, since we expect people coming from more affluent countries to perform more similarly to natives compared to people coming from less developed countries, we distinguish two groups of migrants: those coming from high-income countries (EU15, EFTA, Australia and Oceania, North America, henceforth indicated as HICs) and those coming from high-emigration countries (henceforth HECs).

We define the structure of regional labor market by means of the size of the secondary labor market; we do that by assigning to each region a score that is equal to the share of people employed in low-status jobs, namely those comprised in the first tenth of the ISEI score (such as, for instance, garbage and recycling collectors, cleaners, machine operators, manufacturing laborers).^5^ At the regional level we also observe the share of migrants in the total population, to account for their different distribution among regions, mostly affected by the level of attraction and competitiveness of the local labor market (Ozgen et al., 2012; Lewis and Peri, 2015), and the gross domestic product (GDP) relative change from the previous year,^6^ to consider the economic performance and cycle fluctuations (Kogan, 2006; Dustmann et al., 2010).

We also include individual-level covariates such as the level of education (low: up to lower secondary; mid-high: upper secondary, tertiary or more), age, marital status (single, married/cohabitant, divorced/separated/widowed), and number of children. All continuous variables were standardized in the multilevel analyses.

### Modeling strategy

We use multilevel modeling to predict our two outcomes, namely (1) the probability of being employed and (2) the level of job quality. Our hypotheses postulate a differential effect of the regional labor market for natives and immigrants. In a “simple” regression analysis this would imply interacting the dummy predictors identifying the migrant status (immigrants from HICs and HECs) with the predictor qualifying the labor market (the share of low-quality jobs in the region). However, our data are structured hierarchically, with individuals nested in regions, countries and years of observation. To take this complexity into account, we set up a 3-level model with *N* individuals nested in *J* region/year groups (level-2) and *K* country/year groups (level-3).

We are interested in observing the pattern of employment and job quality as a function of the regional labor market in different subpopulations. Since both labor market dynamics and migration processes are deeply differentiated based on gender, we analyse (A) men and (B) women separately. Furthermore, we are also interested in observing whether the migrant penalty and its regional conditionality plays out differently depending on the respondents' education, hence we run three sets of regressions: (1) for the whole sample, including a predictor for education; (2) for low educated respondents; and (3) for mid-high educated respondents. Finally, to corroborate our expectation of a difference between migrants from HICs and HECs, in the first set of analyses we interact the variables “immigrant from HIC” and “immigrant from HEC” with the region/year-level predictors. All in all, we estimate 16 multilevel regression models, all of which include interactions between regional characteristics and migrants from HECs: two dependent variables (occupational status and job quality), two gender groups (males and females), and three groups defined by education (full sample, low education, mid-high education). Of our two dependent variables, the employment status is binary (the respondent can be either employed or unemployed/inactive) and the level of ISEI index is continuous. Hence, we use a logit model to predict employment status and a linear model to predict job quality.^7^

### Alternative specifications

To explore the scope of our findings we perform several additional checks using modified or even different variables. First, we substitute the share of low-status jobs in a region with a different indicator, the Regional Competitiveness Index (RCI, see Annoni and Dijkstra, 2019). This allows us to see whether our findings hold when using a broader indicator of the concept of labor market quality, which includes also policy-related characteristics. Second, we use a broader definition of the share of low-status jobs at level-2 in our models, looking at those comprised within the first fifth (up to 25.1) instead of the first tenth (up to 17.8) of the ISEI score. This different specification includes in the area of low-skilled jobs occupations like childcare workers, bricklayers, butchers and food preparers, plant operators. Third, we distinguish mid (upper secondary) from high (tertiary or more) educational level, running 4 sets of models (full sample, low education, mid education, high education) instead of 3. This specification allows us to explore with more detail the pattern of the migrant penalty by educational gradients. Fourth, we adopt an alternative operationalization of job quality, using the monthly income from the main job (in deciles of the within-country distribution). By using this indicator, we exclude from the analysis all the self-employed workers as well as the respondents in 159 region/year groups, for whom we miss the information on income. These analyses are shown and discussed in the Supplementary material and are taken into account in the discussion of the findings.

### Descriptive statistics

**Figure 1:**
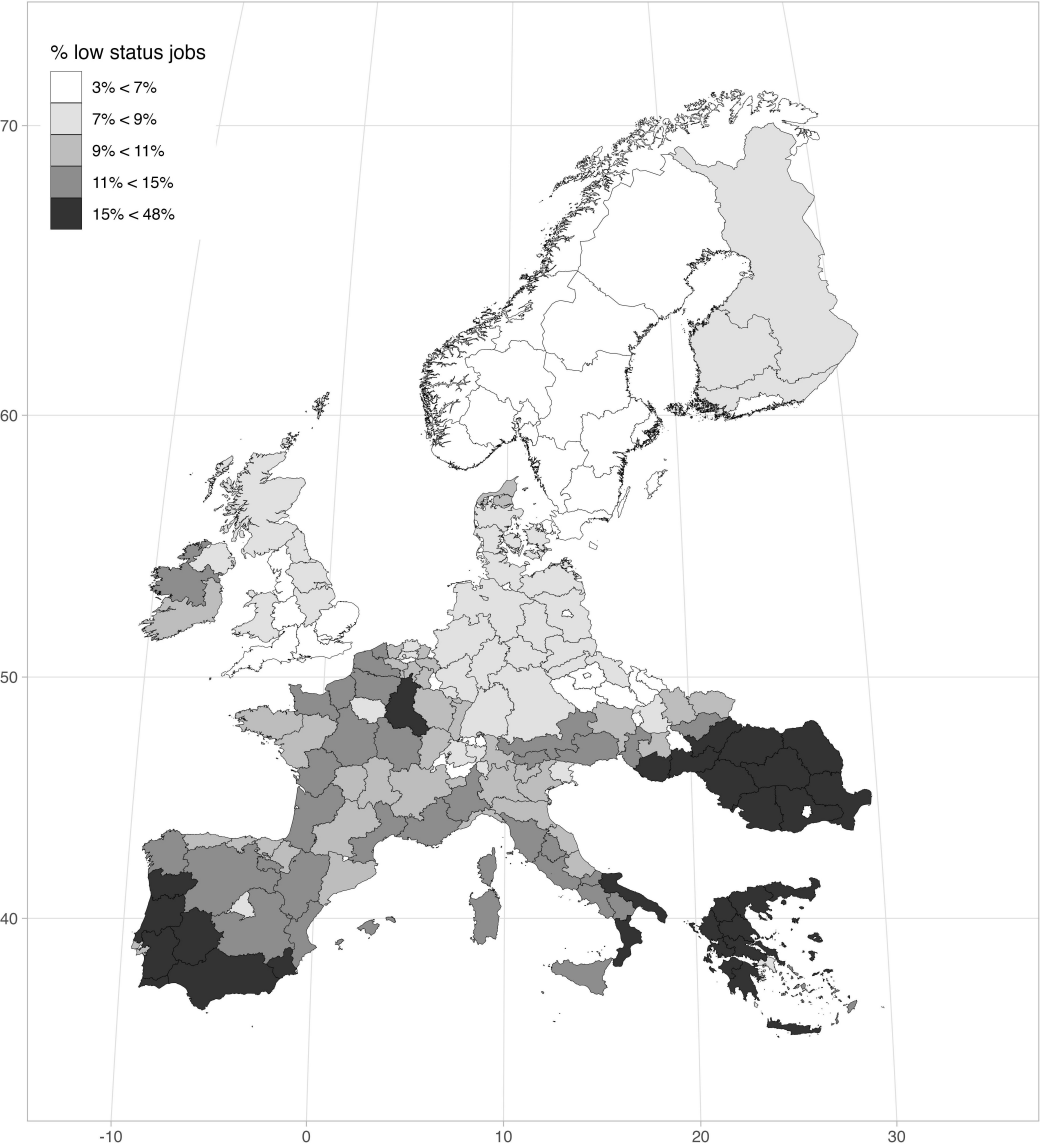
Share of low-status jobs at regional level.

Figure 1 provides visual evidence of how large within-country differences in terms of labor market quality are. The map reports the quintiles in the distribution of the share of low-status jobs at regional level, highlighting a heterogeneous distribution among and within countries: on the one hand, bad jobs are more widespread in Southern European countries and in Romania, while the lowest levels are found in Central-Northern Europe; on the other hand, important internal differences are found in France, Italy, Spain and Hungary. As the figure shows, when differences between regions are considered, country differences appear more blurred, even though most institutional characteristics do not vary within the same country. This confirms the importance of focusing on the regional level when the task is to assess the moderating role of labor markets on our outcomes of interest.

As for other sample characteristics, Supplementary Table S1 reports the main information about it.

## Results

Tables 1, 2 show the results for our two outcomes of interest (respectively, the probability of being employed and the ISEI score). Both tables are structured as follows: the first two columns show the results of the models with the full sample, for males and females, and with the interactions of regional indicators with the “immigrant from HEC” and “immigrant from HIC” variables.^8^

**Table 1:** Multilevel logit models of probability of being employed.

	**Full sample**		**Sample by education**			
	**All females**	**All males**	**Low-edu females**	**Mid-high edu females**	**Low-edu males**	**Mid-high edu males**
Intercept	1.14^***^ (0.02)	1.86^***^ (0.03)	0.15^***^ (0.03)	1.14^***^ (0.02)	1.00^***^ (0.04)	1.83^***^ (0.03)
**Individual-level predictors**						
**Migration status (ref. cat. natives)**						
HEC	−0.80^***^ (0.02)	−0.68^***^ (0.02)	−0.52^***^ (0.03)	−1.00^***^ (0.02)	−0.42^***^ (0.03)	−0.90^***^ (0.02)
HIC	−0.36^***^ (0.02)	−0.11^***^ (0.02)	−0.11^**^ (0.04)	−0.45^***^ (0.02)	0.08 (0.04)	−0.16^***^ (0.02)
Mixed	−0.38^***^ (0.00)	−0.44^***^ (0.00)	−0.25^***^ (0.01)	−0.43^***^ (0.00)	−0.33^***^ (0.01)	−0.46^***^ (0.01)
**Education (ref. cat. mid-high)**						
Low	−0.97^***^ (0.00)	−0.73^***^ (0.00)	–	–	–	–
**Region/year-level predictors**						
Share of low-status jobs	−0.13^***^ (0.01)	−0.11^***^ (0.01)	−0.04^*^ (0.02)	−0.15^***^ (0.01)	0.01 (0.02)	−0.14^***^ (0.01)
Share of migrants	0.15^***^ (0.02)	0.20^***^ (0.02)	0.20^***^ (0.02)	0.13^***^ (0.01)	0.26^***^ (0.02)	0.19^***^ (0.02)
**Cross-level interactions with migrants HEC**						
Share of low-status jobs	0.20^***^ (0.02)	0.18^***^ (0.02)	0.19^***^ (0.03)	0.14^***^ (0.02)	0.23^***^ (0.03)	0.07^**^ (0.02)
Share of migrants	−0.15^***^ (0.03)	−0.35^***^ (0.04)	−0.19^***^ (0.05)	−0.18^***^ (0.03)	−0.25^***^ (0.05)	−0.40^***^ (0.04)
**Cross-level interactions with migrants HIC**						
Share of low-status jobs	−0.09^***^ (0.02)	−0.02 (0.02)	−0.07 (0.04)	−0.08^***^ (0.02)	−0.07 (0.04)	0.01 (0.02)
Share of migrants	0.12^***^ (0.03)	0.04 (0.03)	0.16^**^ (0.06)	0.08^*^ (0.04)	−0.03 (0.06)	0.06 (0.03)
**Observations**						
*N* obs	7,290,190	6,868,990	2,063,711	5,226,479	1,873,331	4,995,659
*N* region/year	1,300	1,300	1,300	1,300	1,300	1,300
*N* country/year	132	132	132	132	132	132

All models control also for: age, family status, number of children, GDP growth at regional level. See the Appendix for the full results. Standard errors between brackets;

*** *p* < 0.001;

** *p* < 0.01;

* *p* < 0.05.

**Table 2:** Multilevel linear models for ISEI score.

	**Full sample**		**Sample by education**			
	**All females**	**All males**	**Low-edu females**	**Mid-high edu females**	**Low-edu males**	**Mid-high edu males**
Intercept	49.54^***^ (0.20)	47.47^***^ (0.28)	28.13^***^ (0.21)	49.96^***^ (0.25)	30.10^***^ (0.18)	48.04^***^ (0.33)
**Individual-level predictors**						
**Migration status (ref. cat. natives)**						
HEC	−11.92^***^ (0.19)	−7.76^***^ (0.16)	−6.62^***^ (0.14)	−15.85^***^ (0.27)	−4.85^***^ (0.14)	−11.45^***^ (0.23)
HIC	1.35^***^ (0.25)	5.64^***^ (0.23)	−1.07^**^ (0.41)	1.69^***^ (0.29)	0.99^**^ (0.34)	6.05^***^ (0.27)
Mixed	−4.23^***^ (0.04)	−3.21^***^ (0.04)	−3.95^***^ (0.06)	−4.60^***^ (0.05)	−2.10^***^ (0.06)	−3.72^***^ (0.05)
**Education (ref. cat. mid-high)**						
Low	−21.87^***^ (0.02)	−17.68^***^ (0.02)	–	–	–	–
**Region/year-level predictors**						
Share of low-status jobs	−2.05^***^ (0.10)	−2.33^***^ (0.10)	−1.98^***^ (0.08)	−1.81^***^ (0.12)	−1.99^***^ (0.07)	−2.12^***^ (0.13)
Share of migrants	2.39^***^ (0.12)	3.81^***^ (0.13)	1.98^***^ (0.11)	2.42^***^ (0.14)	1.99^***^ (0.09)	3.91^***^ (0.16)
**Cross-level interactions with migrants HEC**						
Share of low-status jobs	−1.59^***^ (0.19)	−1.12^***^ (0.15)	1.09^***^ (0.13)	−4.50^***^ (0.27)	0.05 (0.12)	−3.92^***^ (0.24)
Share of migrants	−3.66^***^ (0.30)	−5.56^***^ (0.24)	−2.08^***^ (0.23)	−4.81^***^ (0.41)	−1.91^***^ (0.23)	−7.10^***^ (0.35)
**Cross-level interactions with migrants HIC**						
Share of low-status jobs	−1.61^***^ (0.24)	−0.61^*^ (0.24)	0.76 (0.41)	−2.00^***^ (0.27)	0.71^*^ (0.32)	−0.95^***^ (0.28)
Share of migrants	−1.70^***^ (0.41)	−4.63^***^ (0.35)	−3.36^***^ (0.64)	−1.37^**^ (0.46)	−3.01^***^ (0.53)	−4.49^***^ (0.39)
**Observations**						
*N* obs	4,548,436	5,148,004	858,736	3,689,700	1,174,057	3,973,947
*N* region/year	1,300	1,300	1,300	1,300	1,300	1,300
*N* country/year	132	132	132	132	132	132

All models control also for: age, family status, number of children, GDP growth at regional level. See the Appendix for the full results. Standard errors between brackets;

*** *p* < 0.001;

** *p* < 0.01;

* *p* < 0.05.

Let us first briefly discuss the main effects of the individual-level variables. Migrants coming from less developed countries (HECs) are less likely to be employed in comparison to the natives and, when employed, they have lower quality occupations. This result holds irrespectively for gender and level of education. Differently, migrants from high-income countries (HICs) are less likely to have a job compared to the natives for all groups apart from low-educated males, but, when employed, they tend to have better quality jobs than natives, apart from low-educated females, for whom the effect is negative. Here it emerges a migrant penalty gradient by education and country of origin: the low-educated HICs' migrants are closer to HECs' ones, while the mid-high educated immigrants coming from developed countries are, among the groups of migrants, the most similar to natives. Finally, looking at the “mixed” category, results show that natives without citizenship or citizens who were born abroad tend to perform worse than native citizens on both outcomes.

Focusing on the interactions, the results for the full sample are mixed. Looking at the first two columns of both tables, our trade-off hypothesis seems to be corroborated by the data. Considering the employment status (Table 1), the interaction between the share of low-status jobs in the region and the variable indicating the migrants for HECs is positive and significant for both males (β = 0.18, *p* < 0.001) and females (β = 0.20, *p* < 0.001). This implies that, where the labor market quality is lower, the gap between migrants from HECs and natives in terms of chances to be employed is smaller. This effect holds across alternative specifications of labor market quality (Supplementary Tables S5, S10) and education (Supplementary Table S7). Focusing on the ISEI score (Table 2) the equivalent coefficient is negative for males (β = −1.59, *p* < 0.001) and for females (β = −1.12, *p* < 0.001). These findings suggest an opposite pattern to that observed for employment: the gap in job quality between migrants from HECs and natives increases as the quality of the labor market worsens. This observed association is similar (and arguably stronger) when we look at income instead of the ISEI score as a proxy of job quality (Supplementary Table S9), when we use the RCI as a proxy for labor market quality (Supplementary Table S11), and when labor market quality at meso-level and education are specified differently (Supplementary Tables S6, S8).

If we compare this result with the one obtained by interacting labor market quality with the “migrant from HIC” dummy, we see that the interaction effect goes in the opposite direction in Table 1. This finding implies that migrants from HECs and HICs are very different in terms of chances of being employed: in low-quality labor markets, the former group thrives, while the second is more penalized than the natives.

Another interesting finding regards the difference between the interaction coefficients of migrants from HECs with the share of low-status jobs and the one with the share of immigrants. Looking at Table 1, the interaction coefficients go in opposite directions for both migrants from HECs and HICs. Interestingly, however, the patterns are reversed for these two groups with respect to those observed when interacting the share of low-status jobs in the region. For migrants from less developed countries, living in an area with more immigrants implies having fewer chances to find a job than natives, while for migrants from more developed countries, the chances are higher than for natives. Looking at Table 2, it appears evident that living in regions with more immigrants has a negative impact on the job quality of all migrant workers, who fare worse than natives in all groups.

The results also show that the trade-off effect for this latter group of immigrants is clearly moderated by education (see the last four columns of Tables 1, 2). This pattern is confirmed when labor market quality is operationalized differently (Supplementary Tables S5, S6, S10, S11), and it becomes even more accentuated when we split the level of education into three categories (Supplementary Tables S7, S8). In the models for the probability of being employed, the interaction effect between labor market quality and migrants from HECs is always positive, irrespective of education (Table 1; Supplementary Tables S5, S7, S10). In the models for job quality, the association is highly differentiated depending on the education of the individual. For low-educated individuals, the interaction effect is positive, while for individuals of mid- or high-education it is negative (Table 2; Supplementary Tables S6, S11). Moreover, Supplementary Table S8 shows a further differentiation between individuals of mid and high education, with the association being much stronger for the latter group. Finally, when we look at income (Supplementary Table S9), the interaction effect is negative across all levels of education, however, we note that it is stronger for people of mid and high education, somewhat confirming the pattern observed in the models for ISEI score.

**Figure 2:**
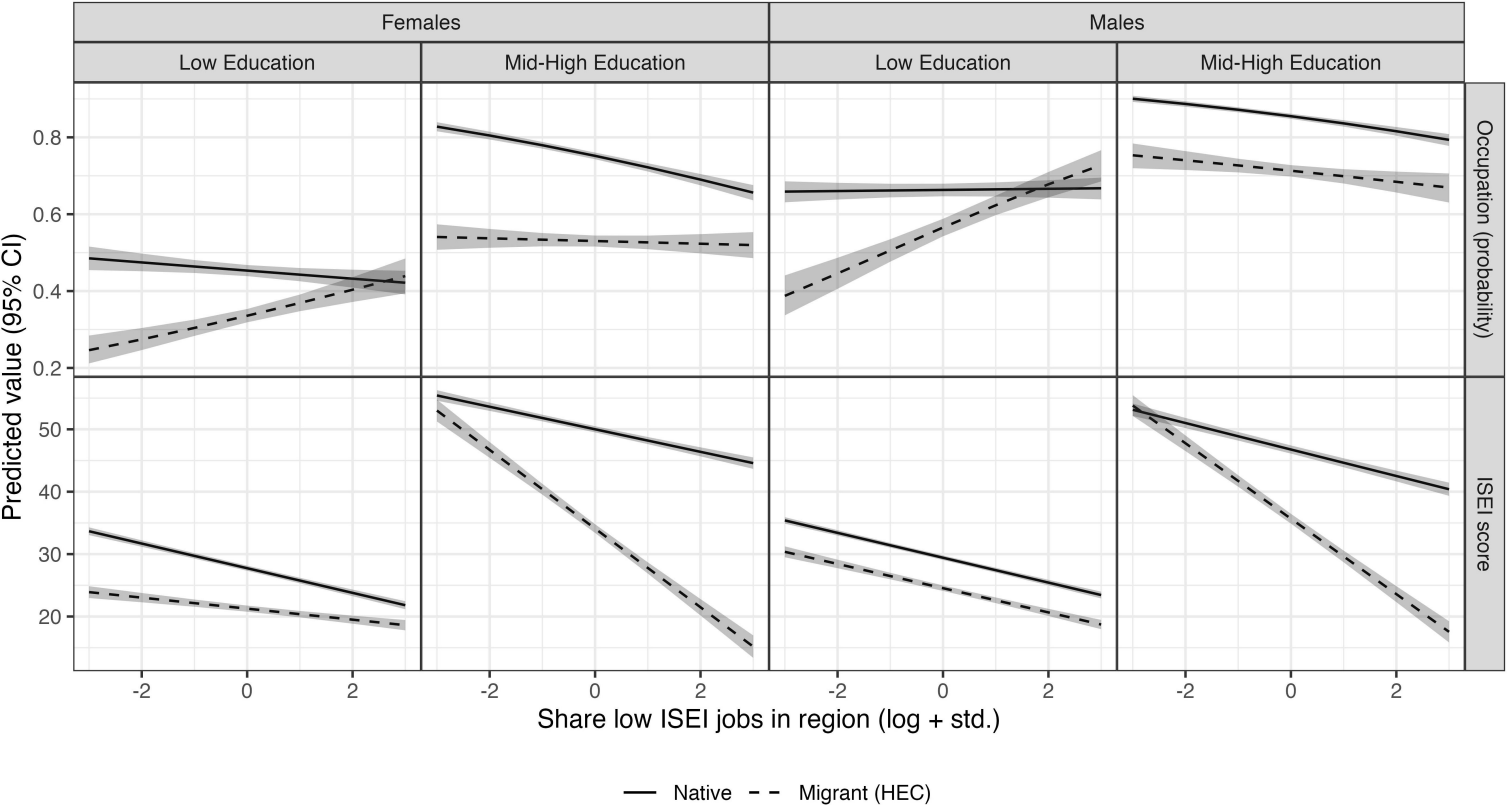
Predicted values of probability of being employed and ISEI score by gender and education.

We offer a further illustration of the patterns observed by means of prediction plots, to address the troublesome interpretability of interaction effects, especially in the case of non-linear models (as in the model for employment) (Mood, 2010). Figure 2 shows the predicted values of the dependent variables (probability of being employed for the first model, ISEI score for the second) for the different subgroups analyzed.

As the figure shows, the difference between natives and migrants shows diverse patterns depending on whether we look at people of low- or mid-high education and, to a lesser extent, males and females. Looking at the probability of being employed (top row), migrants are increasingly better off compared to natives as the share of low-status jobs in the region gets larger. However, this occurs in different ways depending on education. For people of lower education, the probability of having a job is higher for migrants and remains stable for natives. In other words, the quality of the labor market is weakly or not related to the chance that natives have a job, while it is related to the migrants' employment to a great extent: the probability grows from about <0.3 to almost 0.5 for females, and from about 0.4–0.7 for males. This is about 20–30% points (p.p.). If we look at more educated individuals, the scenario is slightly different. Here, the quality of the labor market is weakly associated with the migrants' chances of having a job, while it is associated with the fortunes of natives. As the prevalence of low-status jobs grows, the probability of being employed drops from more than 0.8 to <0.7 for female natives, and from about 0.9 to 0.8 for male natives, namely 10 p.p. for both groups. Unlike with less educated respondents, here migrants are in no place more likely to be employed than natives. In regions with low-quality labor markets migrants and natives are equally likely to have a job, whereas the latter are much better off in places characterized by better-quality labor markets.

Looking at the quality of individual occupations, the picture is much different. The first thing to be noticed is that, across genders and levels of education, natives always land better quality jobs than migrants from less developed countries. The second important point is that, where the labor market quality is worse, the difference between natives and migrants from HECs is larger, but only for people of middle or high education. In regions with higher-quality labor markets, the average ISEI score for natives is about the same as for migrants in the case of both females and males. This difference becomes, respectively, of about 30 and 20 points in regions where the share of low-status occupations is highest. In other words, if we look at more educated individuals, the quality of the labor market is mirrored by a varying difference between natives and migrants, with the latter being more penalized than the former when such quality is worse. Looking at people with lower education, the picture is more similar to what we observed for occupational status. Among the females, the difference between natives and migrants tends to get smaller in worse labor markets. Among the males, the situation is less marked, and the two lines run essentially in parallel.

**Figure 3:**
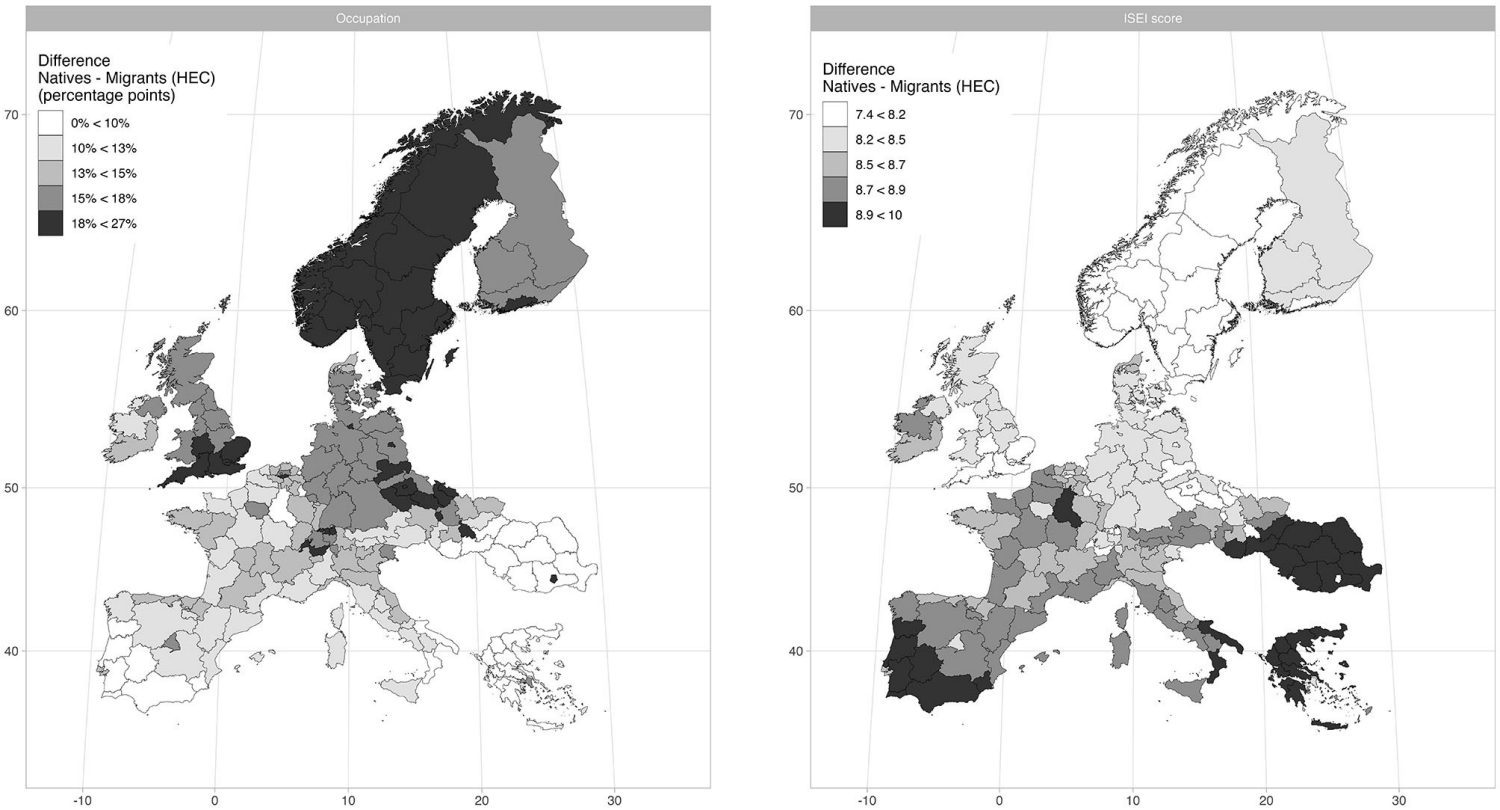
Distribution of predicted value of probability of being employed and ISEI score at regional level.

Just to illustrate the extent of the trade-off between occupation and job quality, Figure 3 replicates the same map shown in Figure 1, but it summarizes the results of the models. In the left panel, the coloring of the regions is based on the quintiles in the distribution of predicted differences of probability of being employed between natives and migrants from HECs (the darker the color, the higher the chance of natives of being employed compared to migrants, and vice versa). As the map shows, in the regions with the lowest share of low-status jobs in Central-Northern Europe (Scandinavia, the south of England, as well as in some German landers, Swiss cantons and Czech regions), natives are much more likely to have a job than immigrants for HECs. In the regions with the highest level of low-status jobs in Southern and Eastern Europe, on the other hand, the difference in chance of being employed between migrants and natives is much smaller, to the point that migrants are sometimes more likely than natives to have a job (Cyprus, Greece, Portugal, Romania, the south of Hungary, Italy and Spain, the Champagne-Ardenne in France). The right panel shows the same exercise done with predicted differences in ISEI score. Here the coloring of the region is essentially reverse. In the same southern and eastern regions, the average ISEI score for migrants from HECs is about 9–10 points lower than natives, whereas in the brighter northern and continental regions such a difference is only around 7–8 points.

## Discussion

This article focuses on the migrant penalty in Europe, measured in terms of probability of being employed and of having a high-quality job, exploring the association with the labor market structure from a regional perspective. With respect to the previous literature on this topic, looking at the regional level allows to improve the accuracy of the analysis. Indeed, it accounts for both the between- and within-country socio-economic and occupational heterogeneity (Hurley et al., 2019; Rosés and Wolf, 2021), reproducing a more detailed outlook of the labor market structure and its “place-based” and “place-specific” institutions and regulation (Tödtling and Trippl, 2005; Iammarino et al., 2019).

We find that the structure of the labor market is strongly associated with different patterns of migrant penalty. In particular, in regions where the share of low-status jobs is higher, we observe a trade-off between the probability of being employed and the quality of the job performed by immigrants from HECs compared to natives (*hypothesis 1 confirmed*). In line with what was observed in Italy (Avola, 2015), in the regions where the share of low-status jobs is higher, migrants are as likely as, or even more likely than natives to be employed, but they face at the same time a higher penalty in terms of ISEI score.

However, the trade-off does not apply *sic et simpliciter* to all migrants. Indeed, worse quality of labor demand at the sub-national level does not correspond to better chances of being employed for HICs' migrants and, among the latter, the penalty for the ISEI is significant only for women (*hypothesis 2 confirmed*). This suggests that the performance of HECs' migrants in the secondary labor markets might be attributed not only to their availability to take the burdens and risks of low-quality jobs, but also to the weaker competition of natives and HICs' immigrants, particularly the highly educated ones, who are less willing to compete for a job in the peripheral area of the occupational structure. It is not a case that, in some regions of Southern and Eastern Europe characterized by a high share of low-status jobs, great immigration from HECs coexists with high levels of unemployment and emigration of natives (often young and educated) with respect to other, more developed regions (Dolado, 2015). From this point of view, the segmented labor theory (Piore, 1979; Massey et al., 1993; Reich, 2008; Auer et al., 2019), emphasizing the relevance of the mismatch between the demand and the supply among natives (but also among HICs' migrants) on the immigrants' labor opportunities and risks, still offers several insights in order to understand the migrant penalty in these new immigrant destinations.

Another interesting finding of our study concerns the heterogeneity of the trade-off effect among HECs' migrants. This trade-off is large for the mid- and high-educated individuals, but it does not occur among the low-educated ones (*hypothesis 3 confirmed*). The former group includes those who, despite having a high probability of having a job, have also more to lose with respect to mid-educated and high-educated natives by working in the peripheral area of the labor market. For low-educated migrants, however, although the employment chances are greater than for low-educated natives in contexts with a high share of low-quality jobs, we observe a “floor effect” on job quality, for which it is almost impossible to perform worse in terms of ISEI score.

Our results suggest that the performance in the host labor market is related to the socio-economic and cultural distance from the natives (Friberg, 2012; Auer et al., 2019), by the difficult transferability of the educational qualifications (Borjas, 1994; Friedberg, 2000; Chiswick and Miller, 2009; Dustmann and Glitz, 2011), without excluding the possibility of different forms of discrimination. In this sense, we observe different degrees of migrant penalty depending on education and country of origin, with the most penalized being the highly educated HECs' immigrants and the most privileged the highly educated HICs' immigrants (the highly skilled globetrotters or “the other side of the moon” of contemporary migration flow, see Mahroum, 2000).

## Conclusion

This paper contributes to the literature on the migrant penalty by exploring how the structure and characteristics of regional labor markets moderate the association between individual features and labor market performances. In particular, we argue that migrants' human capital acts as a valuable resource in the most developed regions, while it does not represent a particular competitive advantage in the least developed areas. On the other hand, if coming from a more socio-economically and culturally distant country can prevent the labor market integration of migrants in the most developed regions in terms both of employment and job quality, the situation is less clear in those regions characterized by large secondary labor markets. In such contexts, immigrants from HICs experience a higher penalty (or a reduced advantage) than elsewhere, while immigrants from HECs fit better with the employers' expectations for jobs considered not suitable for native and HICs' workers. Then, in spite of those who imagine that the less developed regions are not able to absorb immigrant labor force, here the migrants from HECs experience an important advantage with respect to natives in terms of employment opportunity, but they pay it with higher penalty in terms of segregation in the low-status jobs.

Three findings are particularly striking in this study. First, among the institutional characteristics of host societies, the labor market structure plays a decisive role in determining the socio-economic integration of immigrants (Portes and Böröcz, 1989; Reitz, 2002). The regional approach used here improves the focus on a pattern that had been shown by country-level studies. At the same time, the inclusion of a wide variety of countries allows us to maximize the regional variance, offering a truly big picture.

Second, the trade-off effect emerging in the data confirms the great extent to which contemporary migration dynamics are highly differentiated. Far from defining a univocal model of integration, also within the same country, migration flows respond both to the replacement needs of the native supply into the secondary labor markets (the only option in the less developed regions) and to the emergent highly skilled demand expressed by the most dynamic socio-economic contexts.

Third, migrants' human capital is a determinant factor for qualifying the migration experience: on the one hand, highly skilled migrations are limited to the highly educated individuals coming from more affluent countries, whose credentials are recognized and valued; on the other hand, migrants coming from Southern or Eastern areas are segregated in the secondary labor market, irrespective of their educational or professional qualifications.

Jointly taken, all this may reinforce the “ethnic-based” social stratification in less developed European regions; in turn, this may exacerbate the “discontent” in these peripheral areas (Dijkstra et al., 2020), intensifying anti-establishment and anti-immigrant movements and undermining the policies of regional cohesion at the core of the EU political strategy (Garretsen et al., 2013).

Some limitations may be identified in this work. To be sure, our observational design does not allow us to exclude some alternative explanations nor to speak of a direct causal effect of labor markets' characteristics. What this study does is provide a big picture, that is, to show a pattern that is as general as possible, and to interpret it based on what we know from previous studies. The existence of alternative explanations can be investigated by further research, possibly focusing on narrower contexts, or leveraging data that might be available in the future.

Moreover, considering possible further steps on this topic, the same analysis could be conducted considering a longer span of time, testing how the Great Recession has impacted the patterns that emerged in our analysis. Furthermore, the regional perspective could be particularly informative in studying the consequences of the Covid-19 crisis on the labor market structures and workplace location, accounting not only for international migration dynamics but also for internal mobility. Also, it could be interesting to focus on other place-specific indicators, to expand the view on the regional context's characteristics and their association with the migrant penalty. Last, it could be insightful to consider other migrants' characteristics (i.e., legal status, generation) not available in the EU-LFS in order to see how these interact with the socio-economic context.

## Data availability statement

Publicly available datasets were analyzed in this study. This data can be found here: https://ec.europa.eu/eurostat/web/microdata/european-union-labour-force-survey.

## Author contributions

MA: Conceptualization, Data curation, Formal analysis, Funding acquisition, Investigation, Methodology, Resources, Supervision, Writing—original draft. GP: Conceptualization, Data curation, Formal analysis, Investigation, Methodology, Resources, Writing—original draft. FV: Conceptualization, Data curation, Formal analysis, Investigation, Methodology, Resources, Software, Writing—original draft.

## Footnotes

^1^More in detail, Reyneri and Fullin (2011a) highlight that, if protection against firing procedures can influence employers' hiring of immigrants in the primary labor market, this is less relevant in the secondary one, where formal labor regulation can be bypassed by informal rules. Similar results are found by Kogan (2007).

^2^It is paradigmatic, in this regard, the internal divide in Italy between the Centre-North and the South (Avola, 2015), which can be extended to the comparison between the Centre-North and the South of Europe.

^3^We had to exclude from the analysis Bulgaria, Croatia, Malta, Poland, and Slovenia because they don't have any information on respondent country of birth and nationality, Netherlands because of a lack of information at regional level and Cyprus, Estonia, Latvia, Lithuania, and Luxembourg because these countries don't have any regional disaggregation.

^4^The NUTS (Nomenclature of Territorial Units for Statistics) is a hierarchical system for dividing up the EU economic territory (referred to some local administrative units of the member states). It consists of three levels: NUTS1, major socio-economic regions (3–7 million inhabitants); NUTS2, basic regions (800 thousand−3 million); NUTS3, small regions/provinces (150–800 thousand). Although our focus is on the NUTS2 level, information on Austria, Germany and United Kingdom are available only for NUTS1 level.

^5^Similar procedures were used by Kogan (2006), Fleischmann and Dronkers (2010), and Reyneri and Fullin (2011a).

^6^GDP change at national level has been imputed for regions of France, Ireland, and Switzerland, since the indicator at the regional level was missing or not consistent in the period under analysis.

^7^An extended version on our modeling strategy is provided in the Supplementary material.

^8^The tables show only the coefficients of the most relevant predictors. To view the full results, including all covariates, model fit statistics and random effects variances/covariances, see Supplementary Tables S2, S3, where also the alternative specifications are reported and discussed.
