# Supplementary material for: Migrant penalty in the European labor markets: the interplay between individual characteristics and the regional context
Source: Front Sociol. 2023 Nov 1;8:1270167. doi: 10.3389/fsoc.2023.1270167 (PMC10646433; doi:10.3389/fsoc.2023.1270167)
Supplement: Supplementary file 1 [file Table_1.DOCX]

**Migrant Penalty in the European Labour Markets: The Interplay Between Individual Characteristics and the Regional Context**

**Supplemental Material**

**List of tables**

[Table S1: Descriptive statistics 3](#_Toc118560869)

[Table S2: Multilevel logit models of probability of being employed (full results) 5](#_Toc118560870)

[Table S3: Multilevel linear models for ISEI score (full results) 7](#_Toc118560871)

[Table S4: Intraclass correlation coefficients 9](#_Toc118560872)

[Table S5: Multilevel logit models of probability of being employed (using first fifth of the ISEI scale as share of low-status jobs) 13](#_Toc118560873)

[Table S6: Multilevel linear models for ISEI score (using first fifth of the ISEI scale as share of low-status jobs) 15](#_Toc118560874)

[Table S7: Multilevel logit models of probability of being employed (using education in three categories) 17](#_Toc118560875)

[Table S8: Multilevel linear models for ISEI score (using education in three categories) 19](#_Toc118560876)

[Table S9: Multilevel linear models for income from the main job (in deciles) 21](#_Toc118560877)

[Table S10: Multilevel logit models of probability of being employed (using Regional Competitiveness Index instead of share of low-status jobs) 23](#_Toc118560878)

[Table S11: Multilevel linear models for ISEI score (using Regional Competitiveness Index instead of share of low-status jobs) 25](#_Toc118560879)

**Modelling strategy**

We use multilevel modelling to predict our two outcomes, namely (1) the probability of being employed and (2) the level of job quality. Our hypotheses postulate a differential effect of regional labour market for natives and immigrants. In a “simple” regression analysis this would imply interacting the dummy predictors identifying the migrant status (immigrants from HICs and HECs) with the predictor qualifying the labour market (the share of low-quality jobs in the region). However, our data are structured hierarchically, with individuals nested in regions, countries and years of observation. To take this complexity into account, we set up a 3-level model with N individuals nested in J region/year groups (level-2) and K country/year groups (level-3)^[[1]](#footnote-1)^. More formally, we model the outcome Y for an individual i observed in region/year j and country/year k as follows (Gelman and Hill, 2006):

$$Y_{i[j,k]}=\alpha_{0}+\alpha_{j}+\alpha_{k}+{\lambda_{0}F}_{i}+{\lambda_{j}F}_{i}+{\beta X}_{i}+\epsilon_{i}$$

$$\alpha_{j} \sim N({\gamma Z}_{j},\sigma_{j})$$

$$\alpha_{k} \sim N(0,\sigma_{k})$$

$$\lambda_{j} \sim N({\theta W}_{j},\rho_{j})$$

The first equation on top predicts the individual outcome (the probability of being employed or the score of the ISEI index). This is a function of the overall mean (*α_0_*) as well as the mean in the region/year (*α_j_*) and country/year (*α_k_*) where the individual lives, its possible immigrant status (*F_i_*), other individual characteristics (*X_i_*), and a residual error (*ϵ_i_*). The second and third equations define the distribution of the random intercepts among respectively level-2 and level-3 units, that is the average in every region/year and in every country/year unit. We do not have predictors at the country/year-level, hence the intercept *α_k_* is stochastically distributed with mean 0 and residual variance *σ_k_*. On the other hand, we do have predictors at the region/year-level, so we model the intercept *α_j_* as a function of a set of level-2 variables *Z*, with the related fixed coefficients *γ*, and residual variance *σ_j_*. Finally, in the fourth equation, we model the random effect *λ_j_* of being an immigrant (*F_i_*) across region/year units, which is added to the overall fixed effect *λ_0_*. This is a function of our set of level-2 predictors *W*, which includes the share of bad jobs in the region and the share of immigrants in the region as control, as well as the residual variance *ρ_j_*. The fixed coefficients *θ* represent the cross-level interaction effects.

We are interested in observing the pattern of employment and job quality as a function of regional labour market in different subpopulations. Since both labour market dynamics and migration processes are deeply differentiated based on gender, we analyse (A) men and (B) women separately. Furthermore, we are also interested in observing whether the migrant penalty and its regional conditionality plays out differently depending on the respondents’ education, hence we run three sets of regressions: (1) for the whole sample, including a predictor for education; (2) for low educated respondents; and (3) for mid-high educated respondents. Finally, to corroborate our expectation of a difference between migrants from HICs and HECs, in the first set of analyses we interact the variables “immigrant from HIC” and “immigrant from HEC” with the region/year-level predictors. All in all, we estimate 16 multilevel regression models, all of which include interactions between regional characteristics and migrants from HECs: 2 dependent variables (occupational status and job quality), 2 gender groups (males and females), and 3 groups defined by education (full sample, low education, mid-high education). Of our two dependent variables, the employment status is binary (the respondent can be either employed or unemployed) and the level of ISEI index is continuous. Hence, we use a logit model to predict employment status and a linear model to predict job quality.

**Descriptives and main models (full results)**

Table S1 reports the descriptive statistics for the main sample characteristics. The employment rate is close to 70% with a relevant gender gap (13.0%), while the ISEI score mean slightly exceeds 43 points (SD=21.3), highly differentiated by education. Immigrants from HECs represent 4.6% of the whole sample (7.0% among the lowest educated), those from HICs 1,9%, while the residual category 5.3%. Obviously, the share of immigrants at level-2 is highly differentiated, ranging from less than 1% in many regions of Hungary, Romania and Slovak Republic, to more than 50% in Brussels-Capital, Luxembourg and some Swiss cantons. Instead, Tables S2 and S3 replicate Tables 1 and 2 reported in the main text, but including all covariates, model fit statistics and random effects variances/covariances.

**Table S1: Descriptive statistics**

| **Variable** | **Full sample** | **Females** | **Males** | **Low Edu** | **Mid-High Edu** |
| --- | --- | --- | --- | --- | --- |
| Occupational status (employed) | 69.53% (N = 14,159,180) | 63.06% (N = 7,290,190) | 76.4% (N = 6,868,990) | 52.15% (N = 3,937,042) | 76.23% (N = 10,222,138) |
| ISEI score | M = 43.21, SD = 21.27 (N = 9,696,440) | M = 44.73, SD = 21.47 (N = 4,548,436) | M = 41.87, SD = 20.99 (N = 5,148,004) | M = 27.57, SD = 13.2 (N = 2,032,793) | M = 47.36, SD = 21.07 (N = 7,663,647) |
| Migrant (HEC) | 4.59% (N = 14,159,180) | 4.8% (N = 7,290,190) | 4.37% (N = 6,868,990) | 6.99% (N = 3,937,042) | 3.67% (N = 10,222,138) |
| Migrant (HIC) | 1.77% (N = 14,159,180) | 1.7% (N = 7,290,190) | 1.84% (N = 6,868,990) | 1.51% (N = 3,937,042) | 1.87% (N = 10,222,138) |
| Mixed status | 5.23% (N = 14,159,180) | 5.52% (N = 7,290,190) | 4.93% (N = 6,868,990) | 5.34% (N = 3,937,042) | 5.19% (N = 10,222,138) |
| Low Education | 27.81% (N = 14,159,180) | 28.31% (N = 7,290,190) | 27.27% (N = 6,868,990) | - | - |
| Mid Education | 47.25% (N = 14,159,180) | 45.36% (N = 7,290,190) | 49.25% (N = 6,868,990) | - | - |
| High Education | 24.95% (N = 14,159,180) | 26.33% (N = 7,290,190) | 23.48% (N = 6,868,990) | - | - |
| Gender (male) | 48.51% (N = 14,159,180) | - | - | 47.58% (N = 3,937,042) | 48.87% (N = 10,222,138) |
| Age | M = 45.44, SD = 11.08 (N = 14,159,180) | M = 45.57, SD = 11.07 (N = 7,290,190) | M = 45.29, SD = 11.08 (N = 6,868,990) | M = 48.63, SD = 10.73 (N = 3,937,042) | M = 44.21, SD = 10.96 (N = 10,222,138) |
| Age (std.) | M = -0.14, SD = 1 (N = 14,159,180) | M = -0.13, SD = 1 (N = 7,290,190) | M = -0.15, SD = 1 (N = 6,868,990) | M = 0.15, SD = 0.97 (N = 3,937,042) | M = -0.25, SD = 0.99 (N = 10,222,138) |
| Family status: Married/cohabitant | 61.4% (N = 14,159,180) | 62.42% (N = 7,290,190) | 60.31% (N = 6,868,990) | 65.21% (N = 3,937,042) | 59.93% (N = 10,222,138) |
| Family status: Divorced/separated/widowed | 11.27% (N = 14,159,180) | 14.25% (N = 7,290,190) | 8.11% (N = 6,868,990) | 13.31% (N = 3,937,042) | 10.49% (N = 10,222,138) |
| Family status: Single | 27.33% (N = 14,159,180) | 23.33% (N = 7,290,190) | 31.58% (N = 6,868,990) | 21.48% (N = 3,937,042) | 29.59% (N = 10,222,138) |
| Number of children | M = 0.45, SD = 0.83 (N = 14,159,180) | M = 0.46, SD = 0.84 (N = 7,290,190) | M = 0.43, SD = 0.83 (N = 6,868,990) | M = 0.44, SD = 0.86 (N = 3,937,042) | M = 0.45, SD = 0.82 (N = 10,222,138) |
| Share low status jobs in region | 12.11% (N = 14,159,180) | 12.1% (N = 7,290,190) | 12.12% (N = 6,868,990) | 13.93% (N = 3,937,042) | 11.41% (N = 10,222,138) |
| Share low status jobs in region (log & std.) | M = 0.08, SD = 1 (N = 14,159,180) | M = 0.08, SD = 1 (N = 7,290,190) | M = 0.08, SD = 1 (N = 6,868,990) | M = 0.39, SD = 0.93 (N = 3,937,042) | M = -0.04, SD = 1 (N = 10,222,138) |
| Share of migrants in region | 11.72% (N = 14,159,180) | 11.75% (N = 7,290,190) | 11.69% (N = 6,868,990) | 10.87% (N = 3,937,042) | 12.05% (N = 10,222,138) |
| Share of migrants in region (log & std.) | M = -0.07, SD = 1.17 (N = 14,159,180) | M = -0.07, SD = 1.17 (N = 7,290,190) | M = -0.07, SD = 1.17 (N = 6,868,990) | M = -0.06, SD = 1.09 (N = 3,937,042) | M = -0.07, SD = 1.2 (N = 10,222,138) |
| GDP growth in region | M = 0.01, SD = 0.06 (N = 14,159,180) | M = 0.01, SD = 0.06 (N = 7,290,190) | M = 0.01, SD = 0.06 (N = 6,868,990) | M = 0.01, SD = 0.05 (N = 3,937,042) | M = 0.02, SD = 0.06 (N = 10,222,138) |
| GDP growth in region (std.) | M = 0.02, SD = 1.11 (N = 14,159,180) | M = 0.02, SD = 1.11 (N = 7,290,190) | M = 0.02, SD = 1.11 (N = 6,868,990) | M = -0.11, SD = 0.99 (N = 3,937,042) | M = 0.07, SD = 1.14 (N = 10,222,138) |

Note: sample size between brackets

**Table S2: Multilevel logit models of probability of being employed (full results)**

|  | Full sample | | Sample by education | | | |
| --- | --- | --- | --- | --- | --- | --- |
|  | All Females | All Males | Low edu Females | Mid-high edu Females | Low edu Males | Mid-high edu Males |
| Intercept | 1.14 *** | 1.86 *** | 0.15 *** | 1.14 *** | 1.00 *** | 1.83 *** |
|  | (0.02) | (0.03) | (0.03) | (0.02) | (0.04) | (0.03) |
| *Individual-level predictors* | | | | | | |
| Migration status (ref. cat. Natives) | | | | | | |
| HEC | -0.80 *** | -0.68 *** | -0.52 *** | -1.00 *** | -0.42 *** | -0.90 *** |
|  | (0.02) | (0.02) | (0.03) | (0.02) | (0.03) | (0.02) |
| HIC | -0.36 *** | -0.11 *** | -0.11 ** | -0.45 *** | 0.08 | -0.16 *** |
|  | (0.02) | (0.02) | (0.04) | (0.02) | (0.04) | (0.02) |
| Mixed | -0.38 *** | -0.44 *** | -0.25 *** | -0.43 *** | -0.33 *** | -0.46 *** |
|  | (0.00) | (0.00) | (0.01) | (0.00) | (0.01) | (0.01) |
| Education (ref. cat. Mid-high) | | | | | | |
| Low | -0.97 *** | -0.73 *** | - | - | - | - |
|  | (0.00) | (0.00) |  |  |  |  |
| Age | -0.54 *** | -0.69 *** | -0.59 *** | -0.52 *** | -0.64 *** | -0.72 *** |
|  | (0.00) | (0.00) | (0.00) | (0.00) | (0.00) | (0.00) |
| Family status (ref. cat. Married/cohabitant) |  |  |  |  |  |  |
| Divorced/separated/widowed | 0.04 *** | -0.46 *** | 0.09 *** | 0.03 *** | -0.44 *** | -0.46 *** |
|  | (0.00) | (0.00) | (0.00) | (0.00) | (0.01) | (0.00) |
| Single | -0.22 *** | -0.97 *** | -0.25 *** | -0.21 *** | -0.96 *** | -0.96 *** |
|  | (0.00) | (0.00) | (0.00) | (0.00) | (0.00) | (0.00) |
| Number of children | -0.29 *** | 0.20 *** | -0.37 *** | -0.26 *** | 0.08 *** | 0.32 *** |
|  | (0.00) | (0.00) | (0.00) | (0.00) | (0.00) | (0.00) |
| *Region/year-level predictors* | | | | | | |
| Share of low-status jobs | -0.13 *** | -0.11 *** | -0.04 * | -0.15 *** | 0.01 | -0.14 *** |
|  | (0.01) | (0.01) | (0.02) | (0.01) | (0.02) | (0.01) |
| Share of migrants | 0.15 *** | 0.20 *** | 0.20 *** | 0.13 *** | 0.26 *** | 0.19 *** |
|  | (0.02) | (0.02) | (0.02) | (0.01) | (0.02) | (0.02) |
| GDP growth | 0.05 ** | 0.05 ** | 0.04 | 0.05 *** | 0.06 ** | 0.06 *** |
|  | (0.01) | (0.02) | (0.02) | (0.01) | (0.02) | (0.02) |
| *Cross-level interactions with migrants HEC* | | | | | | |
| Share of low-status jobs | 0.20 *** | 0.18 *** | 0.19 *** | 0.14 *** | 0.23 *** | 0.07 ** |
|  | (0.02) | (0.02) | (0.03) | (0.02) | (0.03) | (0.02) |
| Share of migrants | -0.15 *** | -0.35 *** | -0.19 *** | -0.18 *** | -0.25 *** | -0.40 *** |
|  | (0.03) | (0.04) | (0.05) | (0.03) | (0.05) | (0.04) |
| *Cross-level interactions with migrants HIC* | | | | | | |
| Share of low-status jobs | -0.09 *** | -0.02 | -0.07 | -0.08 *** | -0.07 | 0.01 |
|  | (0.02) | (0.02) | (0.04) | (0.02) | (0.04) | (0.02) |
| Share of migrants | 0.12 *** | 0.04 | 0.16 ** | 0.08 * | -0.03 | 0.06 |
|  | (0.03) | (0.03) | (0.06) | (0.04) | (0.06) | (0.03) |
| AIC | 8436476.28 | 6617524.91 | 2565583.44 | 5833126.43 | 2241370.40 | 4343845.04 |
| Log Likelihood | -4218215.14 | -3308739.46 | -1282769.72 | -2916541.22 | -1120663.20 | -2171900.52 |
| N obs | 7,290,190 | 6,868,990 | 2,063,711 | 5,226,479 | 1,873,331 | 4,995,659 |
| N region/year | 1,300 | 1,300 | 1,300 | 1,300 | 1,300 | 1,300 |
| N country/year | 132 | 132 | 132 | 132 | 132 | 132 |
| Var Intercept (r/y) | 0.08 | 0.06 | 0.15 | 0.06 | 0.09 | 0.05 |
| Var Migrant HEC (r/y) | 0.38 | 0.44 | 0.63 | 0.31 | 0.55 | 0.41 |
| Var Migrant HIC (r/y) | 0.14 | 0.13 | 0.20 | 0.13 | 0.26 | 0.13 |
| Var Intercept (c/y) | 0.06 | 0.09 | 0.09 | 0.07 | 0.17 | 0.09 |

Note: standard errors between brackets; *** p < 0.001; ** p < 0.01; * p < 0.05

**Table S3: Multilevel linear models for ISEI score (full results)**

|  | Full sample | | Sample by education | | | |
| --- | --- | --- | --- | --- | --- | --- |
|  | All Females | All Males | Low edu Females | Mid-high edu Females | Low edu Males | Mid-high edu Males |
| Intercept | 49.54 *** | 47.47 *** | 28.13 *** | 49.96 *** | 30.10 *** | 48.04*** |
|  | (0.20) | (0.28) | (0.21) | (0.25) | (0.18) | (0.33) |
| *Individual-level predictors* | | | | | | |
| Migration status (ref. cat. Natives) | | | | | | |
| HEC | -11.92 *** | -7.76 *** | -6.62 *** | -15.85 *** | -4.85 *** | -11.45 *** |
|  | (0.19) | (0.16) | (0.14) | (0.27) | (0.14) | (0.23) |
| HIC | 1.35 *** | 5.64 *** | -1.07 ** | 1.69 *** | 0.99 ** | 6.05 *** |
|  | (0.25) | (0.23) | (0.41) | (0.29) | (0.34) | (0.27) |
| Mixed | -4.23 *** | -3.21 *** | -3.95 *** | -4.60 *** | -2.10 *** | -3.72 *** |
|  | (0.04) | (0.04) | (0.06) | (0.05) | (0.06) | (0.05) |
| Education (ref. cat. Mid-high) | | | | | | |
| Low | -21.87 *** | -17.68 *** | - | - | - | - |
|  | (0.02) | (0.02) |  |  |  |  |
| Age | 0.10 *** | 0.43 *** | -0.52 *** | 0.28 *** | -0.12 *** | 0.62 *** |
|  | (0.01) | (0.01) | (0.02) | (0.01) | (0.02) | (0.01) |
| Family status (ref. cat. Married/cohabitant) |  |  |  |  |  |  |
| Divorced/separated/widowed | -1.56 *** | -2.20 *** | -0.28 *** | -1.97 *** | -0.26 *** | -2.94 *** |
|  | (0.03) | (0.03) | (0.04) | (0.03) | (0.04) | (0.04) |
| Single | 0.88 *** | -2.10 *** | -0.26 *** | 0.96 *** | -2.23 *** | -2.23 *** |
|  | (0.02) | (0.02) | (0.04) | (0.03) | (0.03) | (0.03) |
| Number of children | 0.19 *** | 0.11 *** | -0.30 *** | 0.24 *** | -0.22 *** | 0.14 *** |
|  | (0.01) | (0.01) | (0.02) | (0.02) | (0.01) | (0.01) |
| *Region/year-level predictors* | | | | | | |
| Share of low-status jobs | -2.05 *** | -2.33 *** | -1.98 *** | -1.81 *** | -1.99 *** | -2.12 *** |
|  | (0.10) | (0.10) | (0.08) | (0.12) | (0.07) | (0.13) |
| Share of migrants | 2.39 *** | 3.81 *** | 1.98 *** | 2.42 *** | 1.99 *** | 3.91 *** |
|  | (0.12) | (0.13) | (0.11) | (0.14) | (0.09) | (0.16) |
| GDP growth | -0.10 | 0.02 | 0.24^*^ | -0.19 | 0.07 | -0.08 |
|  | (0.12) | (0.13) | (0.11) | (0.14) | (0.09) | (0.17) |
| *Cross-level interactions with migrants HEC* | | | | | | |
| Share of low-status jobs | -1.59 *** | -1.12 *** | 1.09 *** | -4.50 *** | 0.05 | -3.92 *** |
|  | (0.19) | (0.15) | (0.13) | (0.27) | (0.12) | (0.24) |
| Share of migrants | -3.66 *** | -5.56 *** | -2.08 *** | -4.81 *** | -1.91 *** | -7.10 *** |
|  | (0.30) | (0.24) | (0.23) | (0.41) | (0.23) | (0.35) |
| *Cross-level interactions with migrants HIC* | | | | | | |
| Share of low-status jobs | -1.61 *** | -0.61 * | 0.76 | -2.00 *** | 0.71 * | -0.95 *** |
|  | (0.24) | (0.24) | (0.41) | (0.27) | (0.32) | (0.28) |
| Share of migrants | -1.70 *** | -4.63 *** | -3.36 *** | -1.37 ** | -3.01 *** | -4.49 *** |
|  | (0.41) | (0.35) | (0.64) | (0.46) | (0.53) | (0.39) |
| AIC | 39627563.44 | 44867956.71 | 6783216.54 | 32555075.39 | 9250052.59 | 35213378.78 |
| Log Likelihood | -19813757.72 | -22433954.36 | -3391585.27 | -16277514.70 | -4625003.30 | -17606666.39 |
| N obs | 4548436 | 5148004 | 858736 | 3689700 | 1174057 | 3973947 |
| N region/year | 1300 | 1300 | 1300 | 1300 | 1300 | 1300 |
| N country/year | 132 | 132 | 132 | 132 | 132 | 132 |
| Var Intercept (r/y) | 3.07 | 3.50 | 2.02 | 4.38 | 1.27 | 5.31 |
| Var Migrant HEC (r/y) | 27.44 | 16.64 | 5.33 | 56.15 | 5.69 | 42.90 |
| Var Migrant HIC (r/y) | 20.06 | 24.59 | 37.20 | 23.98 | 26.58 | 29.40 |
| Var Intercept (c/y) | 4.62 | 9.47 | 5.38 | 7.73 | 4.13 | 13.83 |

Note: standard errors between brackets; *** p < 0.001; ** p < 0.01; * p < 0.05

**Intraclass Correlation Coefficients**

**Table S4: Intraclass correlation coefficients**

| ICC level | Employment Status (F) | Employment Status (M) | ISEI score (F) | ISEI score (M) |
| --- | --- | --- | --- | --- |
| Country/year | 5.08% | 3.31% | 3.15% | 4.06% |
| Region/year | 1.9% | 1.7% | 1.84% | 2.36% |

In Table S4, we report the intraclass correlation coefficients (ICCs) of the four main models (Employment status and ISEI score, males and females). The ICC reflects the percentage of variance in the empty model (a model without predictors) that is attributed to the variation between groups, and can be defined as the proportion of the variance of the intercepts at the region/year and country/year levels over the total variance in the empty model. In the two logit models for employment status, the ICC was calculated as follows:

$${ICC}_{region/year}= \frac{\sigma_{j}}{\sigma_{j}+\sigma_{k}+ \frac{\pi^{2}}{3}}$$

$${ICC}_{country/year}= \frac{\sigma_{k}}{\sigma_{j}+\sigma_{k}+ \frac{\pi^{2}}{3}}$$

In the two linear models for ISEI score, the ICCs were calculated as follows:

$${ICC}_{region/year}= \frac{\sigma_{j}}{\sigma_{j}+\sigma_{k}+ \sigma_{i}}$$

$${ICC}_{country/year}= \frac{\sigma_{k}}{\sigma_{j}+\sigma_{k}+ \sigma_{i}}$$

Where *σ_j_* is the variance of the region/year-level intercepts and *σ_k_* is the variance of the country/year-level intercepts. In the linear models for ISEI score, the total variance includes the residual variance *σ_i_*. In the logit models for employment status, the residual variance is fixed at *π^2^/3*.

**Robustness checks**

We performed several additional analyses to those reported in the paper in Tables 1 and 2. Tables S5 and S6 replicate the models partially reported in the text and fully reported in Tables S2 and S3, with one difference: to determine the share of low-status jobs in a region, we do not consider all the jobs in the first *tenth* of the ISEI scale as we do in the main analyses, but we consider the jobs in the first *fifth* of the scale. As a result, we include in the share a wider range of jobs, obtaining in general higher shares across the regions. The results are substantively identical to those reported in the text for what concerns the models for employment status (Table S5), while the interaction effects between the share of low-status jobs and the “migrant from HEC” dummy become nonsignificant in the models on full samples (first 2 columns of Table S6). When we split the sample in two further groups by level of education, all the patterns remain the same as in the analyses reported in the paper.

Tables S7 and S8 also replicate the models reported in Tables S2 and S3, but this time we coded educational levels in three categories (low, middle, high) instead of two (low, middle-high). Here, too, the results for employment status remain largely consistent with those reported in the paper. With respect to ISEI score the interaction effects between the share of low-status jobs and the “migrant from HEC” dummy become nonsignificant for males in the models on full samples (second column of Table S8), while the pattern of coefficients when splitting respondents by educational level becomes even more nuanced than the results reported in the paper, underlining a negative gradient of the effect when moving from highly educated people to low educated ones

Table S9 reports a sort of conceptual replication of the models for ISEI score, using a different outcome variable: the respondent’s income from the main job. These models are based on a different population as the models for ISEI score, as income is not observed in all countries in the LFS data and, more importantly, it is not asked to self-employed respondents. However, the results replicate substantively what is reported in the models for ISEI score.

Tables S10 and S11 replicate again the analyses reported in Tables S2 and S3, with a crucial difference: instead of the “share of low-status jobs” we use as indicator of labour market quality the Regional Competitiveness Index (RCI), an indicator developed by the Regional and Urban Policy section of the European Commission to measure “the ability of a region to offer an attractive and sustainable environment for firms and residents to live and work” (Annoni and Dijkstra 2019, p. 3). The RCI is a composite index consisting of 11 “pillars”, reflecting different measured aspects of regional competitiveness: (1) Institutions; (2) Macroeconomic Stability; (3) Infrastructures; (4) Health; (5) Basic Education; (6) Higher Education, Training and Lifelong Learning; (7) Labour Market Efficiency; (8) Market Size; (9) Technological Readiness; (10) Business Sophistication; (11) Innovation. These pillars are measured using 74 indicators, some observed at the country level (i.e. the indicators for Macroeconomic Stability, Basic Education, and some indicators for Institutions and Technological Readiness). See Annoni and Dijkstra (2019) for a thorough discussion of the methodology to compute the index and its validity. As Figure S1 shows, the RCI index correlates well with the share of low ISEI jobs, showing that the latter measure represents the quality of regional labour markets well. The results reported in Tables S10 and S11 show that the RCI works as well as the share of low-status jobs (albeit with reversed sign, being an indicator of “good quality” rather than “bad quality” of the labour market).

In further robustness checks, we introduce two country-level policy indicators, the EPL index (strictness of employment protection) and the MIPEX index (favourability of immigration policies), and interact them with the indicator of migrants from HECs. This allows us to control for characteristics that are constant between regions of the same country but vary considerably from one country to another. Additionally, we replicate all the analyses on two different subsamples if individuals, dividing short-term migrants (who have been living in the host country for less than 10 years) and long-term migrants, and including a dummy variable identifying the respondents born abroad but in an EU-member state. In all these specifications, available on request from the authors, the interactions effects with the regional-level indicators that we include in the paper remain similar, confirming the robustness of our findings.


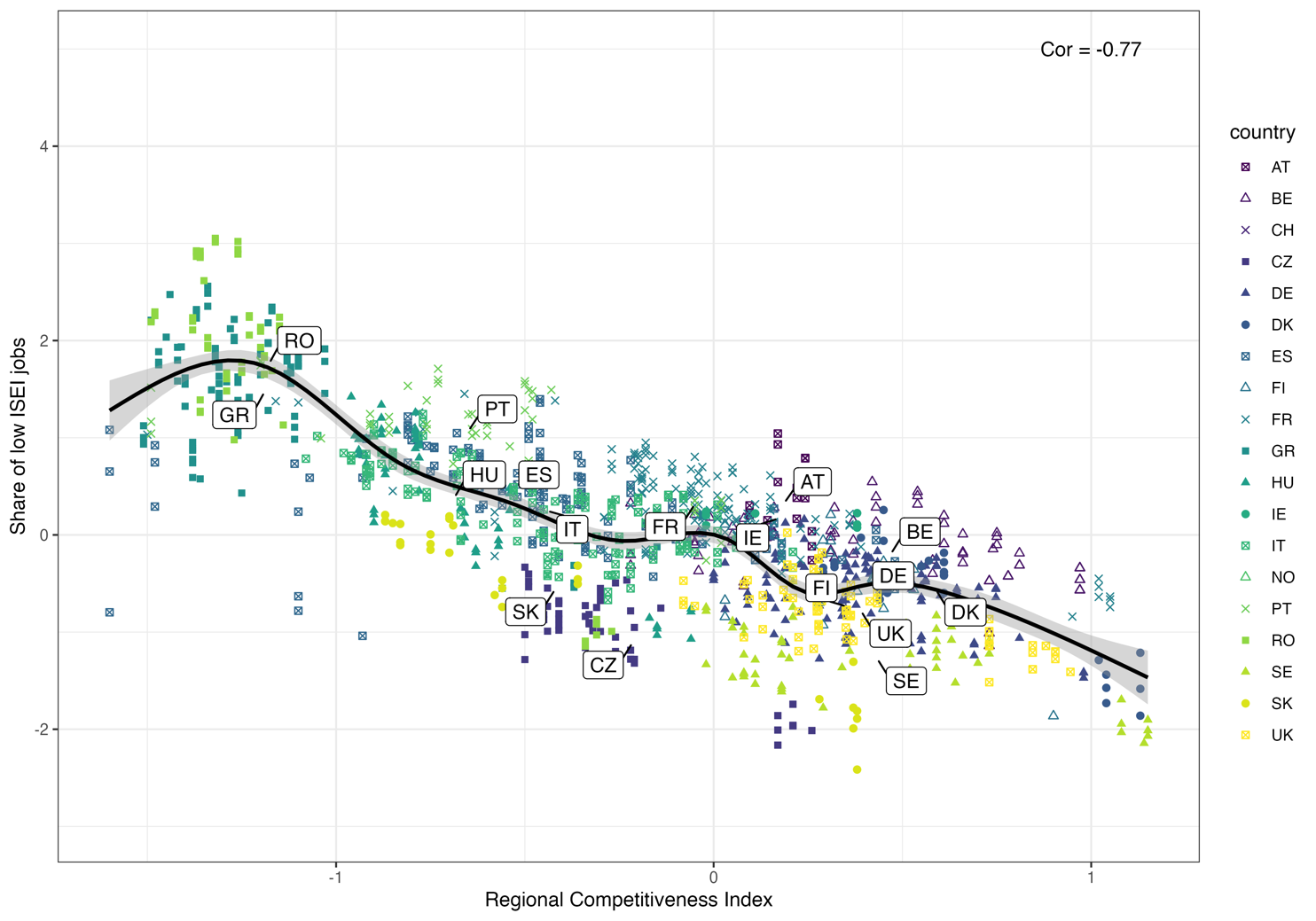


**Figure S1: Correlation between share of low ISEI jobs and Regional Competitiveness Index**

**Table S5: Multilevel logit models of probability of being employed (using first fifth of the ISEI scale as share of low-status jobs)**

|  | Full sample | | Sample by education | | | |
| --- | --- | --- | --- | --- | --- | --- |
|  | All Females | All Males | Lower ed. Females | Mid-high ed. Females | Lower ed. Males | Mid-high ed. Males |
| Intercept | 1.15^***^ | 1.87^***^ | 0.16^***^ | 1.15^***^ | 1.01^***^ | 1.84^***^ |
|  | (0.02) | (0.03) | (0.03) | (0.03) | (0.04) | (0.03) |
| *Individual-level predictors* | | | | | | |
| Migration status (ref. cat. Natives) | | | | | | |
| HEC | -0.80^***^ | -0.68^***^ | -0.51^***^ | -1.00^***^ | -0.41^***^ | -0.90^***^ |
|  | (0.02) | (0.02) | (0.03) | (0.02) | (0.03) | (0.02) |
| HIC | -0.36^***^ | -0.11^***^ | -0.10^**^ | -0.44^***^ | 0.10^*^ | -0.16^***^ |
|  | (0.02) | (0.02) | (0.04) | (0.02) | (0.04) | (0.02) |
| Mixed | -0.38^***^ | -0.44^***^ | -0.25^***^ | -0.43^***^ | -0.33^***^ | -0.46^***^ |
|  | (0.00) | (0.00) | (0.01) | (0.00) | (0.01) | (0.01) |
| Education (ref. cat. Mid-high) | | | | | | |
| Low | -0.97^***^ | -0.73^***^ | - | - | - | - |
|  | (0.00) | (0.00) |  |  |  |  |
| Age | -0.54^***^ | -0.69^***^ | -0.59^***^ | -0.52^***^ | -0.64^***^ | -0.72^***^ |
|  | (0.00) | (0.00) | (0.00) | (0.00) | (0.00) | (0.00) |
| Family status (ref. cat. Married/cohabitant) |  |  |  |  |  |  |
| Divorced/separated/widowed | 0.04^***^ | -0.46^***^ | 0.09^***^ | 0.03^***^ | -0.44^***^ | -0.46^***^ |
|  | (0.00) | (0.00) | (0.00) | (0.00) | (0.01) | (0.00) |
| Single | -0.22^***^ | -0.97^***^ | -0.25^***^ | -0.21^***^ | -0.96^***^ | -0.96^***^ |
|  | (0.00) | (0.00) | (0.00) | (0.00) | (0.00) | (0.00) |
| Number of children | -0.29^***^ | 0.20^***^ | -0.37^***^ | -0.26^***^ | 0.08^***^ | 0.32^***^ |
|  | (0.00) | (0.00) | (0.00) | (0.00) | (0.00) | (0.00) |
| *Region/year-level predictors* | | | | | | |
| Share of low-status jobs | -0.06^***^ | -0.05^***^ | 0.03 | -0.08^***^ | 0.06^***^ | -0.08^***^ |
|  | (0.01) | (0.01) | (0.02) | (0.01) | (0.02) | (0.01) |
| Share of migrants | 0.16^***^ | 0.22^***^ | 0.23^***^ | 0.15^***^ | 0.30^***^ | 0.20^***^ |
|  | (0.02) | (0.02) | (0.02) | (0.02) | (0.02) | (0.02) |
| GDP growth | 0.05^***^ | 0.06^***^ | 0.04^*^ | 0.06^***^ | 0.06^**^ | 0.06^***^ |
|  | (0.02) | (0.02) | (0.02) | (0.02) | (0.02) | (0.02) |
| *Cross-level interactions with migrants HEC* | | | | | | |
| Share of low-status jobs | 0.12^***^ | 0.13^***^ | 0.09^**^ | 0.08^***^ | 0.16^***^ | 0.02 |
|  | (0.02) | (0.03) | (0.03) | (0.02) | (0.03) | (0.03) |
| Share of migrants | -0.15^***^ | -0.34^***^ | -0.21^***^ | -0.18^***^ | -0.24^***^ | -0.42^***^ |
|  | (0.03) | (0.04) | (0.05) | (0.03) | (0.06) | (0.04) |
| *Cross-level interactions with migrants HIC* | | | | | | |
| Share of low-status jobs | -0.10^***^ | -0.04 | -0.08^*^ | -0.09^***^ | -0.11^**^ | -0.01 |
|  | (0.02) | (0.02) | (0.04) | (0.02) | (0.04) | (0.02) |
| Share of migrants | 0.09^*^ | 0.02 | 0.12 | 0.04 | -0.10 | 0.05 |
|  | (0.04) | (0.03) | (0.07) | (0.04) | (0.07) | (0.04) |
| AIC | 8436576.80 | 6617599.00 | 2565609.21 | 5833250.69 | 2241380.12 | 4343934.85 |
| Log Likelihood | -4218265.40 | -3308776.50 | -1282782.60 | -2916603.35 | -1120668.06 | -2171945.42 |
| N obs | 7290190 | 6868990 | 2063711 | 5226479 | 1873331 | 4995659 |
| N region/year | 1300 | 1300 | 1300 | 1300 | 1300 | 1300 |
| N country/year | 132 | 132 | 132 | 132 | 132 | 132 |
| Var Intercept (r/y) | 0.09 | 0.06 | 0.15 | 0.06 | 0.09 | 0.05 |
| Var Migrant HEC (r/y) | 0.40 | 0.47 | 0.67 | 0.33 | 0.59 | 0.42 |
| Var Migrant HIC (r/y) | 0.14 | 0.13 | 0.19 | 0.13 | 0.26 | 0.13 |
| Var Intercept (c/y) | 0.06 | 0.10 | 0.10 | 0.08 | 0.17 | 0.11 |

Note: standard errors between brackets; *** p < 0.001; ** p < 0.01; * p < 0.05

**Table S6: Multilevel linear models for ISEI score (using first fifth of the ISEI scale as share of low-status jobs)**

|  | Full sample | | Sample by education | | | |
| --- | --- | --- | --- | --- | --- | --- |
|  | All Females | All Males | Low edu Females | Mid-high edu Females | Low edu Males | Mid-high edu Males |
| Intercept | 49.43^***^ | 47.39^***^ | 28.14^***^ | 49.83^***^ | 30.11^***^ | 47.90^***^ |
|  | (0.19) | (0.24) | (0.21) | (0.26) | (0.17) | (0.32) |
| *Individual-level predictors* | | | | | | |
| Migration status (ref. cat. Natives) | | | | | | |
| HEC | -11.87^***^ | -7.75^***^ | -6.78^***^ | -15.62^***^ | -4.90^***^ | -11.30^***^ |
|  | (0.20) | (0.16) | (0.14) | (0.28) | (0.14) | (0.24) |
| HIC | 1.49^***^ | 5.65^***^ | -0.96^*^ | 1.83^***^ | 1.10^**^ | 6.08^***^ |
|  | (0.26) | (0.23) | (0.42) | (0.29) | (0.34) | (0.27) |
| Mixed | -4.23^***^ | -3.21^***^ | -3.96^***^ | -4.60^***^ | -2.11^***^ | -3.72^***^ |
|  | (0.04) | (0.04) | (0.06) | (0.05) | (0.06) | (0.05) |
| Education (ref. cat. Mid-high) | | | | | | |
| Low | -21.87^***^ | -17.67^***^ | - | - | - | - |
|  | (0.02) | (0.02) |  |  |  |  |
| Age | 0.10^***^ | 0.43^***^ | -0.52^***^ | 0.28^***^ | -0.12^***^ | 0.62^***^ |
|  | (0.01) | (0.01) | (0.02) | (0.01) | (0.02) | (0.01) |
| Family status (ref. cat. Married/cohabitant) |  |  |  |  |  |  |
| Divorced/separated/widowed | -1.56^***^ | -2.20^***^ | -0.28^***^ | -1.97^***^ | -0.26^***^ | -2.94^***^ |
|  | (0.03) | (0.03) | (0.04) | (0.03) | (0.04) | (0.04) |
| Single | 0.88^***^ | -2.10^***^ | -0.26^***^ | 0.96^***^ | -2.23^***^ | -2.23^***^ |
|  | (0.02) | (0.02) | (0.04) | (0.03) | (0.03) | (0.03) |
| Number of children | 0.19^***^ | 0.11^***^ | -0.30^***^ | 0.24^***^ | -0.22^***^ | 0.14^***^ |
|  | (0.01) | (0.01) | (0.02) | (0.02) | (0.01) | (0.01) |
| *Region/year-level predictors* | | | | | | |
| Share of low-status jobs | -2.77^***^ | -3.00^***^ | -2.09^***^ | -2.62^***^ | -1.93^***^ | -2.99^***^ |
|  | (0.07) | (0.08) | (0.08) | (0.10) | (0.06) | (0.11) |
| Share of migrants | 1.40^***^ | 2.70^***^ | 1.40^***^ | 1.37^***^ | 1.56^***^ | 2.71^***^ |
|  | (0.10) | (0.12) | (0.11) | (0.14) | (0.10) | (0.15) |
| GDP growth | -0.11 | 0.02 | 0.25^*^ | -0.19 | 0.13 | -0.08 |
|  | (0.10) | (0.11) | (0.10) | (0.13) | (0.08) | (0.14) |
| *Cross-level interactions with migrants HEC* | | | | | | |
| Share of low-status jobs | -1.26^***^ | -0.83^***^ | 1.50^***^ | -4.01^***^ | 0.16 | -3.44^***^ |
|  | (0.21) | (0.17) | (0.14) | (0.30) | (0.14) | (0.27) |
| Share of migrants | -4.03^***^ | -5.73^***^ | -1.41^***^ | -6.01^***^ | -1.92^***^ | -8.00^***^ |
|  | (0.33) | (0.27) | (0.25) | (0.46) | (0.26) | (0.39) |
| *Cross-level interactions with migrants HIC* | | | | | | |
| Share of low-status jobs | -1.61^***^ | -0.77^**^ | 0.13 | -1.92^***^ | 0.62 | -1.05^***^ |
|  | (0.25) | (0.25) | (0.42) | (0.28) | (0.33) | (0.29) |
| Share of migrants | -2.36^***^ | -4.87^***^ | -3.80^***^ | -2.09^***^ | -3.15^***^ | -4.81^***^ |
|  | (0.45) | (0.37) | (0.73) | (0.50) | (0.60) | (0.42) |
| AIC | 39627026.37 | 44867434.78 | 6783113.56 | 32554759.08 | 9250050.58 | 35213039.54 |
| Log Likelihood | -19813489.18 | -22433693.39 | -3391533.78 | -16277356.54 | -4625002.29 | -17606496.77 |
| N obs | 4548436 | 5148004 | 858736 | 3689700 | 1174057 | 3973947 |
| N region/year | 1300 | 1300 | 1300 | 1300 | 1300 | 1300 |
| N country/year | 132 | 132 | 132 | 132 | 132 | 132 |
| Var Intercept (r/y) | 1.70 | 2.10 | 1.69 | 2.87 | 1.16 | 3.52 |
| Var Migrant HEC (r/y) | 28.90 | 17.54 | 4.97 | 62.50 | 6.12 | 47.47 |
| Var Migrant HIC (r/y) | 20.06 | 24.39 | 37.32 | 24.24 | 27.02 | 29.28 |
| Var Intercept (c/y) | 4.46 | 7.32 | 5.27 | 8.43 | 3.64 | 12.80 |

Note: standard errors between brackets; *** p < 0.001; ** p < 0.01; * p < 0.05

**Table S7: Multilevel logit models of probability of being employed (using education in three categories)**

|  | All Females | All Males | Low edu Females | Mid edu  Females | High edu Females | Low edu Males | Mid edu  Males | High edu Males |
| --- | --- | --- | --- | --- | --- | --- | --- | --- |
| Intercept | 0.94^***^ | 1.71^***^ | 0.15^***^ | 0.96^***^ | 1.53^***^ | 1.00^***^ | 1.70^***^ | 2.12^***^ |
|  | (0.02) | (0.03) | (0.03) | (0.02) | (0.03) | (0.04) | (0.03) | (0.03) |
| *Individual-level predictors* | | | | | | | | |
| Migration status (ref. cat. Natives) | | | | | | | | |
| HEC | -0.81^***^ | -0.69^***^ | -0.52^***^ | -0.79^***^ | -1.31^***^ | -0.42^***^ | -0.79^***^ | -1.16^***^ |
|  | (0.02) | (0.02) | (0.03) | (0.02) | (0.03) | (0.03) | (0.03) | (0.03) |
| HIC | -0.42^***^ | -0.17^***^ | -0.11^**^ | -0.50^***^ | -0.54^***^ | 0.08 | -0.29^***^ | -0.20^***^ |
|  | (0.02) | (0.02) | (0.04) | (0.03) | (0.03) | (0.04) | (0.03) | (0.04) |
| Mixed | -0.39^***^ | -0.45^***^ | -0.25^***^ | -0.39^***^ | -0.57^***^ | -0.33^***^ | -0.47^***^ | -0.52^***^ |
|  | (0.00) | (0.00) | (0.01) | (0.01) | (0.01) | (0.01) | (0.01) | (0.01) |
| Education (ref. cat. Mid) | | | | | | | | |
| Low | -0.77^***^ | -0.59^***^ | - | - | - | - | - | - |
|  | (0.00) | (0.00) |  |  |  |  |  |  |
| High | 0.65^***^ | 0.49^***^ | - | - | - | - | - | - |
|  | (0.00) | (0.00) |  |  |  |  |  |  |
| Age | -0.53^***^ | -0.68^***^ | -0.59^***^ | -0.59^***^ | -0.35^***^ | -0.64^***^ | -0.78^***^ | -0.56^***^ |
|  | (0.00) | (0.00) | (0.00) | (0.00) | (0.00) | (0.00) | (0.00) | (0.00) |
| Family status (ref. cat. Married/cohabitant) |  |  |  |  |  |  |  |  |
| Divorced/separated/widowed | 0.06^***^ | -0.45^***^ | 0.09^***^ | 0.05^***^ | 0.08^***^ | -0.44^***^ | -0.45^***^ | -0.38^***^ |
|  | (0.00) | (0.00) | (0.00) | (0.00) | (0.01) | (0.01) | (0.00) | (0.01) |
| Single | -0.25^***^ | -0.97^***^ | -0.25^***^ | -0.27^***^ | -0.16^***^ | -0.96^***^ | -0.97^***^ | -0.89^***^ |
|  | (0.00) | (0.00) | (0.00) | (0.00) | (0.00) | (0.00) | (0.00) | (0.01) |
| Number of children | -0.29^***^ | 0.20^***^ | -0.37^***^ | -0.36^***^ | -0.10^***^ | 0.08^***^ | 0.25^***^ | 0.51^***^ |
|  | (0.00) | (0.00) | (0.00) | (0.00) | (0.00) | (0.00) | (0.00) | (0.00) |
| *Region/year-level predictors* |  |  |  |  |  |  |  |  |
| Share of low-status jobs | -0.12^***^ | -0.09^***^ | -0.04^*^ | -0.15^***^ | -0.10^***^ | 0.01 | -0.12^***^ | -0.15^***^ |
|  | (0.01) | (0.01) | (0.02) | (0.01) | (0.01) | (0.02) | (0.01) | (0.01) |
| Share of migrants | 0.13^***^ | 0.18^***^ | 0.20^***^ | 0.11^***^ | 0.08^***^ | 0.26^***^ | 0.16^***^ | 0.13^***^ |
|  | (0.02) | (0.02) | (0.02) | (0.02) | (0.02) | (0.02) | (0.02) | (0.02) |
| GDP growth | 0.05^**^ | 0.05^**^ | 0.04 | 0.05^**^ | 0.05^***^ | 0.06^**^ | 0.06^***^ | 0.05^**^ |
|  | (0.01) | (0.02) | (0.02) | (0.02) | (0.02) | (0.02) | (0.02) | (0.02) |
| *Cross-level interactions with migrants HEC* |  |  |  |  |  |  |  |  |
| Share of low-status jobs | 0.22^***^ | 0.20^***^ | 0.19^***^ | 0.19^***^ | 0.08^**^ | 0.23^***^ | 0.06^*^ | 0.13^***^ |
|  | (0.02) | (0.02) | (0.03) | (0.02) | (0.03) | (0.03) | (0.03) | (0.03) |
| Share of migrants | -0.14^***^ | -0.32^***^ | -0.19^***^ | -0.12^***^ | -0.23^***^ | -0.25^***^ | -0.37^***^ | -0.18^***^ |
|  | (0.03) | (0.04) | (0.05) | (0.03) | (0.04) | (0.05) | (0.04) | (0.05) |
| *Cross-level interactions with migrants HIC* |  |  |  |  |  |  |  |  |
| Share of low-status jobs | -0.07^***^ | -0.01 | -0.07 | -0.08^*^ | -0.09^***^ | -0.07 | 0.01 | 0.01 |
|  | (0.02) | (0.02) | (0.04) | (0.03) | (0.03) | (0.04) | (0.03) | (0.03) |
| Share of migrants | 0.11^**^ | 0.07^*^ | 0.16^**^ | 0.15^**^ | -0.06 | -0.03 | 0.16^***^ | 0.05 |
|  | (0.04) | (0.03) | (0.06) | (0.05) | (0.05) | (0.06) | (0.04) | (0.05) |
| AIC | 8354582.58 | 6586475.20 | 2565583.44 | 3922897.61 | 1806328.85 | 2241370.40 | 3152174.89 | 1149510.15 |
| Log Likelihood | -4177267.29 | -3293213.60 | -1282769.72 | -1961426.80 | -903142.43 | -1120663.20 | -1576065.44 | -574733.07 |
| N obs | 7290190 | 6868990 | 2063711 | 3306748 | 1919731 | 1873331 | 3382834 | 1612825 |
| N region/year | 1300 | 1300 | 1300 | 1300 | 1300 | 1300 | 1300 | 1300 |
| N country/year | 132 | 132 | 132 | 132 | 132 | 132 | 132 | 132 |
| Var Intercept (r/y) | 0.08 | 0.06 | 0.15 | 0.08 | 0.04 | 0.09 | 0.06 | 0.04 |
| Var Migrant HEC (r/y) | 0.41 | 0.46 | 0.63 | 0.32 | 0.40 | 0.55 | 0.44 | 0.44 |
| Var Migrant HIC (r/y) | 0.16 | 0.13 | 0.20 | 0.26 | 0.17 | 0.26 | 0.13 | 0.23 |
| Var Intercept (c/y) | 0.06 | 0.09 | 0.09 | 0.06 | 0.10 | 0.17 | 0.09 | 0.12 |

Note: standard errors between brackets; *** p < 0.001; ** p < 0.01; * p < 0.05

**Table S8: Multilevel linear models for ISEI score (using education in three categories)**

|  | All Females | All Males | Low edu Females | Mid edu  Females | High edu Females | Low edu Males | Mid edu  Males | High edu Males |
| --- | --- | --- | --- | --- | --- | --- | --- | --- |
| Intercept | 40.02^***^ | 38.43^***^ | 28.13^***^ | 40.03^***^ | 65.41^***^ | 30.10^***^ | 38.99^***^ | 64.59^***^ |
|  | (0.20) | (0.20) | (0.21) | (0.26) | (0.32) | (0.18) | (0.22) | (0.37) |
| *Individual-level predictors* | | | | | | | | |
| Migration status (ref. cat. Natives) | | | | | | | | |
| HEC | -11.09^***^ | -7.45^***^ | -6.62^***^ | -13.02^***^ | -18.85^***^ | -4.85^***^ | -9.49^***^ | -15.22^***^ |
|  | (0.16) | (0.14) | (0.14) | (0.21) | (0.39) | (0.14) | (0.17) | (0.39) |
| HIC | -0.82^***^ | 1.85^***^ | -1.07^**^ | -0.54 | -1.64^***^ | 0.99^**^ | 2.07^***^ | 0.46 |
|  | (0.20) | (0.17) | (0.41) | (0.33) | (0.29) | (0.34) | (0.29) | (0.26) |
| Mixed | -4.45^***^ | -3.15^***^ | -3.95^***^ | -4.82^***^ | -5.08^***^ | -2.10^***^ | -3.66^***^ | -4.16^***^ |
|  | (0.03) | (0.03) | (0.06) | (0.05) | (0.06) | (0.06) | (0.05) | (0.07) |
| Education (ref. cat. Mid) | | | | | | | | |
| Low | -12.53^***^ | -9.02^***^ | - | - | - | - | - | - |
|  | (0.02) | (0.02) |  |  |  |  |  |  |
| High | 23.89^***^ | 25.20^***^ | - | - | - | - | - | - |
|  | (0.02) | (0.02) |  |  |  |  |  |  |
| Age | 0.88^***^ | 0.68^***^ | -0.52^***^ | 0.84^***^ | 1.66^***^ | -0.12^***^ | 0.66^***^ | 1.43^***^ |
|  | (0.01) | (0.01) | (0.02) | (0.01) | (0.02) | (0.02) | (0.01) | (0.02) |
| Family status (ref. cat. Married/cohabitant) |  |  |  |  |  |  |  |  |
| Divorced/separated/widowed | -0.87^***^ | -1.12^***^ | -0.28^***^ | -0.71^***^ | -1.77^***^ | -0.26^***^ | -1.22^***^ | -2.15^***^ |
|  | (0.02) | (0.03) | (0.04) | (0.03) | (0.05) | (0.04) | (0.04) | (0.07) |
| Single | -0.14^***^ | -1.75^***^ | -0.26^***^ | 0.16^***^ | -0.62^***^ | -2.23^***^ | -2.05^***^ | -1.18^***^ |
|  | (0.02) | (0.02) | (0.04) | (0.03) | (0.03) | (0.03) | (0.03) | (0.04) |
| Number of children | -0.04^***^ | -0.09^***^ | -0.30^***^ | -0.30^***^ | 0.14^***^ | -0.22^***^ | -0.24^***^ | 0.25^***^ |
|  | (0.01) | (0.01) | (0.02) | (0.02) | (0.02) | (0.01) | (0.01) | (0.02) |
| *Region/year-level predictors* |  |  |  |  |  |  |  |  |
| Share of low-status jobs | -1.34^***^ | -1.48^***^ | -1.98^***^ | -1.35^***^ | -0.05 | -1.99^***^ | -1.30^***^ | -0.61^***^ |
|  | (0.07) | (0.07) | (0.08) | (0.10) | (0.09) | (0.07) | (0.09) | (0.11) |
| Share of migrants | 2.06^***^ | 2.61^***^ | 1.98^***^ | 2.75^***^ | 1.01^***^ | 1.99^***^ | 3.13^***^ | 1.38^***^ |
|  | (0.09) | (0.09) | (0.11) | (0.13) | (0.12) | (0.09) | (0.12) | (0.15) |
| GDP growth | -0.08 | -0.01 | 0.24^*^ | -0.09 | -0.08 | 0.07 | -0.03 | 0.00 |
|  | (0.09) | (0.09) | (0.11) | (0.13) | (0.12) | (0.09) | (0.12) | (0.15) |
| *Cross-level interactions with migrants HEC* |  |  |  |  |  |  |  |  |
| Share of low-status jobs | -0.51^**^ | -0.17 | 1.09^***^ | -1.87^***^ | -5.66^***^ | 0.05 | -1.34^***^ | -5.43^***^ |
|  | (0.16) | (0.14) | (0.13) | (0.21) | (0.41) | (0.12) | (0.17) | (0.42) |
| Share of migrants | -3.06^***^ | -3.36^***^ | -2.08^***^ | -3.16^***^ | -4.01^***^ | -1.91^***^ | -3.71^***^ | -4.60^***^ |
|  | (0.25) | (0.21) | (0.23) | (0.32) | (0.61) | (0.23) | (0.26) | (0.56) |
| *Cross-level interactions with migrants HIC* |  |  |  |  |  |  |  |  |
| Share of low-status jobs | -0.94^***^ | 0.00 | 0.76 | -0.97^***^ | -1.85^***^ | 0.71^*^ | 0.02 | -0.53^*^ |
|  | (0.18) | (0.18) | (0.41) | (0.29) | (0.26) | (0.32) | (0.29) | (0.26) |
| Share of migrants | -2.15^***^ | -2.79^***^ | -3.36^***^ | -1.83^***^ | -0.50 | -3.01^***^ | -3.59^***^ | -0.16 |
|  | (0.31) | (0.26) | (0.64) | (0.50) | (0.45) | (0.53) | (0.41) | (0.36) |
| AIC | 38058947.65 | 43099364.18 | 6783216.54 | 18227596.11 | 12819302.80 | 9250052.59 | 21761386.40 | 11781597.09 |
| Log Likelihood | -19029448.83 | -21549657.09 | -3391585.27 | -9113775.05 | -6409628.40 | -4625003.30 | -10880670.20 | -5890775.54 |
| N obs | 4548436 | 5148004 | 858736 | 2172576 | 1517124 | 1174057 | 2609974 | 1363973 |
| N region/year | 1300 | 1300 | 1300 | 1300 | 1300 | 1300 | 1300 | 1300 |
| N country/year | 132 | 132 | 132 | 132 | 132 | 132 | 132 | 132 |
| Var Intercept (r/y) | 1.47 | 1.60 | 2.02 | 3.27 | 1.95 | 1.27 | 3.03 | 2.97 |
| Var Migrant HEC (r/y) | 18.42 | 13.14 | 5.33 | 26.01 | 122.94 | 5.69 | 16.66 | 117.43 |
| Var Migrant HIC (r/y) | 10.32 | 11.01 | 37.20 | 23.95 | 18.24 | 26.58 | 25.23 | 15.40 |
| Var Intercept (c/y) | 5.26 | 4.73 | 5.38 | 8.38 | 13.17 | 4.13 | 6.14 | 17.86 |

Note: standard errors between brackets; *** p < 0.001; ** p < 0.01; * p < 0.05

**Table S9: Multilevel linear models for income from the main job (in deciles)**

|  | Full sample | | Sample by education | | | |
| --- | --- | --- | --- | --- | --- | --- |
|  | All Females | All Males | Low edu Females | Mid-high edu Females | Lower edu Males | Mid-high edu Males |
| Intercept | 5.36^***^ | 7.19^***^ | 3.21^***^ | 5.43^***^ | 5.45^***^ | 7.22^***^ |
|  | (0.08) | (0.07) | (0.06) | (0.09) | (0.07) | (0.08) |
| *Individual-level predictors* | | | | | | |
| Migration status (ref. cat. Natives) | | | | | | |
| HEC | -1.23^***^ | -1.39^***^ | -0.70^***^ | -1.70^***^ | -1.21^***^ | -1.69^***^ |
|  | (0.03) | (0.02) | (0.03) | (0.04) | (0.04) | (0.03) |
| HIC | -0.12^**^ | 0.19^***^ | -0.08 | -0.19^***^ | -0.05 | 0.22^***^ |
|  | (0.04) | (0.04) | (0.06) | (0.05) | (0.06) | (0.04) |
| Mixed | -0.35^***^ | -0.58^***^ | -0.18^***^ | -0.46^***^ | -0.30^***^ | -0.66^***^ |
|  | (0.01) | (0.01) | (0.01) | (0.01) | (0.01) | (0.01) |
| Education (ref. cat. Mid-high) | | | | | | |
| Low | -2.22^***^ | -1.62^***^ | - | - | - | - |
|  | (0.00) | (0.00) |  |  |  |  |
| Age | 0.34^***^ | 0.26^***^ | 0.10^***^ | 0.39^***^ | 0.17^***^ | 0.29^***^ |
|  | (0.00) | (0.00) | (0.00) | (0.00) | (0.00) | (0.00) |
| Family status (ref. cat. Married/cohabitant) |  |  |  |  |  |  |
| Divorced/separated/widowed | 0.15^***^ | -0.49^***^ | 0.17^***^ | 0.15^***^ | -0.45^***^ | -0.50^***^ |
|  | (0.00) | (0.01) | (0.01) | (0.01) | (0.01) | (0.01) |
| Single | 0.38^***^ | -0.87^***^ | 0.19^***^ | 0.41^***^ | -1.02^***^ | -0.82^***^ |
|  | (0.00) | (0.00) | (0.01) | (0.00) | (0.01) | (0.00) |
| Number of children | -0.11^***^ | 0.19^***^ | -0.11^***^ | -0.12^***^ | 0.14^***^ | 0.21^***^ |
|  | (0.00) | (0.00) | (0.00) | (0.00) | (0.00) | (0.00) |
| *Region/year-level predictors* | | | | | | |
| Share of low-status jobs | -0.31^***^ | -0.29^***^ | -0.26^***^ | -0.30^***^ | -0.27^***^ | -0.26^***^ |
|  | (0.02) | (0.02) | (0.02) | (0.02) | (0.03) | (0.02) |
| Share of migrants | 0.37^***^ | 0.61^***^ | 0.30^***^ | 0.36^***^ | 0.48^***^ | 0.60^***^ |
|  | (0.03) | (0.03) | (0.03) | (0.03) | (0.04) | (0.03) |
| GDP growth | -0.02 | 0.04 | 0.02 | -0.03 | 0.04 | 0.03 |
|  | (0.03) | (0.03) | (0.03) | (0.03) | (0.04) | (0.03) |
| *Cross-level interactions with migrants HEC* | | | | | | |
| Share of low-status jobs | -0.34^***^ | -0.28^***^ | -0.03 | -0.70^***^ | -0.24^***^ | -0.52^***^ |
|  | (0.03) | (0.03) | (0.03) | (0.04) | (0.03) | (0.03) |
| Share of migrants | -0.13^**^ | -0.54^***^ | 0.05 | -0.24^***^ | -0.03 | -0.62^***^ |
|  | (0.05) | (0.04) | (0.04) | (0.06) | (0.06) | (0.04) |
| *Cross-level interactions with migrants HIC* | | | | | | |
| Share of low-status jobs | -0.27^***^ | -0.13^**^ | -0.09 | -0.36^***^ | -0.09 | -0.16^***^ |
|  | (0.05) | (0.04) | (0.06) | (0.05) | (0.06) | (0.05) |
| Share of migrants | 0.16^*^ | -0.28^***^ | -0.28^***^ | 0.28^***^ | -0.13 | -0.27^***^ |
|  | (0.07) | (0.05) | (0.08) | (0.07) | (0.08) | (0.05) |
| AIC | 12403678.92 | 12572791.92 | 2019167.95 | 10298408.58 | 2688187.46 | 9864432.82 |
| Log Likelihood | -6201815.46 | -6286371.96 | -1009560.97 | -5149181.29 | -1344070.73 | -4932193.41 |
| N obs | 2648907 | 2771797 | 480766 | 2168141 | 603422 | 2168375 |
| N region/year | 1141 | 1141 | 1141 | 1141 | 1141 | 1141 |
| N country/year | 113 | 113 | 113 | 113 | 113 | 113 |
| Var Intercept (r/y) | 0.10 | 0.14 | 0.09 | 0.11 | 0.20 | 0.14 |
| Var Migrant HEC (r/y) | 0.50 | 0.32 | 0.15 | 0.84 | 0.28 | 0.44 |
| Var Migrant HIC (r/y) | 0.34 | 0.33 | 0.17 | 0.38 | 0.12 | 0.36 |
| Var Intercept (c/y) | 0.67 | 0.59 | 0.37 | 0.84 | 0.54 | 0.64 |

Note: standard errors between brackets; *** p < 0.001; ** p < 0.01; * p < 0.05

**Table S10: Multilevel logit models of probability of being employed (using Regional Competitiveness Index instead of share of low-status jobs)**

|  | Full sample | | Sample by education | | | |
| --- | --- | --- | --- | --- | --- | --- |
|  | All Females | All Males | Low edu Females | Mid-high edu Females | Lower edu Males | Mid-high edu Males |
| Intercept | 1.17^***^ | 1.83^***^ | 0.15^***^ | 1.17^***^ | 0.97^***^ | 1.79^***^ |
|  | (0.02) | (0.03) | (0.03) | (0.02) | (0.04) | (0.02) |
| *Individual-level predictors* | | | | | | |
| Migration status (ref. cat. Natives) | | | | | | |
| HEC | -0.90^***^ | -0.77^***^ | -0.61^***^ | -1.07^***^ | -0.52^***^ | -0.94^***^ |
|  | (0.02) | (0.02) | (0.03) | (0.02) | (0.03) | (0.03) |
| HIC | -0.35^***^ | -0.11^***^ | -0.07 | -0.45^***^ | 0.05 | -0.16^***^ |
|  | (0.02) | (0.02) | (0.04) | (0.02) | (0.05) | (0.03) |
| Mixed | -0.38^***^ | -0.44^***^ | -0.24^***^ | -0.44^***^ | -0.33^***^ | -0.46^***^ |
|  | (0.00) | (0.00) | (0.01) | (0.00) | (0.01) | (0.01) |
| Education (ref. cat. Mid-high) | | | | | | |
| Low | -0.98^***^ | -0.73^***^ | - | - | - | - |
|  | (0.00) | (0.00) |  |  |  |  |
| Age | -0.54^***^ | -0.69^***^ | -0.60^***^ | -0.53^***^ | -0.64^***^ | -0.72^***^ |
|  | (0.00) | (0.00) | (0.00) | (0.00) | (0.00) | (0.00) |
| Family status (ref. cat. Married/cohabitant) |  |  |  |  |  |  |
| Divorced/separated/widowed | 0.04^***^ | -0.46^***^ | 0.09^***^ | 0.02^***^ | -0.44^***^ | -0.45^***^ |
|  | (0.00) | (0.00) | (0.00) | (0.00) | (0.01) | (0.00) |
| Single | -0.24^***^ | -0.97^***^ | -0.25^***^ | -0.23^***^ | -0.96^***^ | -0.95^***^ |
|  | (0.00) | (0.00) | (0.00) | (0.00) | (0.00) | (0.00) |
| Number of children | -0.29^***^ | 0.21^***^ | -0.37^***^ | -0.26^***^ | 0.09^***^ | 0.32^***^ |
|  | (0.00) | (0.00) | (0.00) | (0.00) | (0.00) | (0.00) |
| *Region/year-level predictors* | | | | | | |
| Regional Competitiveness Index | 0.46^***^ | 0.31^***^ | 0.46^***^ | 0.42^***^ | 0.26^***^ | 0.34^***^ |
|  | (0.02) | (0.02) | (0.03) | (0.02) | (0.03) | (0.02) |
| Share of migrants | 0.03^*^ | 0.11^***^ | 0.04 | 0.05^**^ | 0.15^***^ | 0.10^***^ |
|  | (0.02) | (0.02) | (0.02) | (0.01) | (0.02) | (0.02) |
| GDP growth | 0.03^*^ | 0.03^*^ | 0.01 | 0.04^**^ | 0.04 | 0.04^**^ |
|  | (0.01) | (0.02) | (0.02) | (0.01) | (0.02) | (0.01) |
| *Cross-level interactions with migrants HEC* | | | | | | |
| Regional Competitiveness Index | -0.57^***^ | -0.54^***^ | -0.55^***^ | -0.45^***^ | -0.58^***^ | -0.31^***^ |
|  | (0.03) | (0.04) | (0.05) | (0.03) | (0.05) | (0.04) |
| Share of migrants | 0.01 | -0.19^***^ | -0.02 | -0.03 | -0.05 | -0.33^***^ |
|  | (0.03) | (0.04) | (0.06) | (0.04) | (0.06) | (0.04) |
| *Cross-level interactions with migrants HIC* | | | | | | |
| Regional Competitiveness Index | 0.14^***^ | 0.07 | 0.23^***^ | 0.07 | 0.14 | 0.03 |
|  | (0.04) | (0.04) | (0.06) | (0.04) | (0.07) | (0.05) |
| Share of migrants | 0.05 | 0.03 | -0.00 | 0.06 | -0.02 | 0.05 |
|  | (0.04) | (0.04) | (0.08) | (0.05) | (0.09) | (0.04) |
| AIC | 8177349.55 | 6456498.16 | 2515089.27 | 5625557.35 | 2206996.19 | 4217311.22 |
| Log Likelihood | -4088651.77 | -3228226.08 | -1257522.63 | -2812756.67 | -1103476.10 | -2108633.61 |
| N obs | 7038576 | 6635629 | 2025223 | 5013353 | 1841355 | 4794274 |
| N region/year | 1190 | 1190 | 1190 | 1190 | 1190 | 1190 |
| N country/year | 118 | 118 | 118 | 118 | 118 | 118 |
| Var Intercept (r/y) | 0.07 | 0.05 | 0.15 | 0.05 | 0.09 | 0.04 |
| Var Migrant HEC (r/y) | 0.34 | 0.42 | 0.62 | 0.29 | 0.54 | 0.42 |
| Var Migrant HIC (r/y) | 0.16 | 0.15 | 0.24 | 0.16 | 0.32 | 0.17 |
| Var Intercept (c/y) | 0.04 | 0.06 | 0.09 | 0.04 | 0.16 | 0.05 |

Note: standard errors between brackets; *** p < 0.001; ** p < 0.01; * p < 0.05

**Table S11: Multilevel linear models for ISEI score (using Regional Competitiveness Index instead of share of low-status jobs)**

|  | Full sample | | Sample by education | | | |
| --- | --- | --- | --- | --- | --- | --- |
|  | All Females | All Males | Low edu Females | Mid-high edu Females | Lower edu Males | Mid-high edu Males |
| Intercept | 49.86^***^ | 47.86^***^ | 28.16^***^ | 50.29^***^ | 30.23^***^ | 48.42^***^ |
|  | (0.18) | (0.26) | (0.20) | (0.25) | (0.15) | (0.34) |
| *Individual-level predictors* | | | | | | |
| Migration status (ref. cat. Natives) | | | | | | |
| HEC | -11.36^***^ | -7.24^***^ | -6.98^***^ | -14.42^***^ | -4.69^***^ | -10.12^***^ |
|  | (0.20) | (0.16) | (0.16) | (0.27) | (0.16) | (0.23) |
| HIC | 1.62^***^ | 5.87^***^ | -1.56^***^ | 2.12^***^ | 0.83^*^ | 6.38^***^ |
|  | (0.28) | (0.24) | (0.44) | (0.31) | (0.37) | (0.28) |
| Mixed | -4.38^***^ | -3.28^***^ | -3.91^***^ | -4.82^***^ | -2.08^***^ | -3.86^***^ |
|  | (0.04) | (0.04) | (0.06) | (0.05) | (0.06) | (0.05) |
| Education (ref. cat. Mid-high) | | | | | | |
| Low | -21.92^***^ | -17.66^***^ | - | - | - | - |
|  | (0.02) | (0.02) |  |  |  |  |
| Age | 0.15^***^ | 0.46^***^ | -0.51^***^ | 0.34^***^ | -0.12^***^ | 0.65^***^ |
|  | (0.01) | (0.01) | (0.02) | (0.01) | (0.02) | (0.01) |
| Family status (ref. cat. Married/cohabitant) |  |  |  |  |  |  |
| Divorced/separated/widowed | -1.61^***^ | -2.22^***^ | -0.36^***^ | -2.01^***^ | -0.30^***^ | -2.97^***^ |
|  | (0.03) | (0.03) | (0.04) | (0.03) | (0.05) | (0.04) |
| Single | 0.81^***^ | -2.13^***^ | -0.29^***^ | 0.90^***^ | -2.25^***^ | -2.26^***^ |
|  | (0.02) | (0.02) | (0.04) | (0.03) | (0.03) | (0.03) |
| Number of children | 0.20^***^ | 0.11^***^ | -0.30^***^ | 0.24^***^ | -0.22^***^ | 0.14^***^ |
|  | (0.01) | (0.01) | (0.02) | (0.02) | (0.01) | (0.01) |
| *Region/year-level predictors* | | | | | | |
| Regional Competitiveness Index | 2.01^***^ | 3.60^***^ | 2.38^***^ | 1.56^***^ | 2.53^***^ | 3.37^***^ |
|  | (0.20) | (0.21) | (0.16) | (0.25) | (0.14) | (0.26) |
| Share of migrants | 2.48^***^ | 3.43^***^ | 1.97^***^ | 2.68^***^ | 1.96^***^ | 3.58^***^ |
|  | (0.14) | (0.15) | (0.12) | (0.17) | (0.10) | (0.18) |
| GDP growth | -0.06 | -0.03 | 0.26^*^ | -0.15 | 0.08 | -0.13 |
|  | (0.12) | (0.14) | (0.11) | (0.15) | (0.09) | (0.17) |
| *Cross-level interactions with migrants HEC* | | | | | | |
| Regional Competitiveness Index | 3.57^***^ | 2.48^***^ | -2.18^***^ | 9.38^***^ | -0.13 | 8.48^***^ |
|  | (0.32) | (0.26) | (0.20) | (0.45) | (0.20) | (0.40) |
| Share of migrants | -6.09^***^ | -7.31^***^ | -1.87^***^ | -9.26^***^ | -2.86^***^ | -10.78^***^ |
|  | (0.35) | (0.28) | (0.27) | (0.46) | (0.29) | (0.39) |
| *Cross-level interactions with migrants HIC* | | | | | | |
| Regional Competitiveness Index | 2.01^***^ | 0.13 | -2.99^***^ | 3.31^***^ | -2.57^***^ | 1.14^*^ |
|  | (0.44) | (0.47) | (0.75) | (0.50) | (0.61) | (0.55) |
| Share of migrants | -2.68^***^ | -4.78^***^ | -1.01 | -2.89^***^ | -1.63^*^ | -5.04^***^ |
|  | (0.53) | (0.44) | (0.87) | (0.60) | (0.72) | (0.49) |
| AIC | 38089670.05 | 43219283.09 | 6606561.99 | 31197958.53 | 9077605.71 | 33743976.10 |
| Log Likelihood | -19044811.02 | -21609617.55 | -3303257.99 | -15598956.27 | -4538779.86 | -16871965.05 |
| N obs | 4372764 | 4962630 | 837145 | 3535619 | 1153059 | 3809571 |
| N region/year | 1190 | 1190 | 1190 | 1190 | 1190 | 1190 |
| N country/year | 118 | 118 | 118 | 118 | 118 | 118 |
| Var Intercept (r/y) | 3.95 | 3.62 | 2.46 | 5.20 | 1.73 | 5.37 |
| Var Migrant HEC (r/y) | 24.76 | 15.41 | 5.08 | 47.19 | 6.28 | 35.77 |
| Var Migrant HIC (r/y) | 23.21 | 27.41 | 40.82 | 27.37 | 31.14 | 33.38 |
| Var Intercept (c/y) | 3.11 | 7.19 | 4.06 | 6.33 | 2.26 | 12.30 |

Note: standard errors between brackets; *** p < 0.001; ** p < 0.01; * p < 0.05

**References**

Annoni, Paola, and Lewis Dijkstra. 2019. “The EU Regional Competitiveness Index 2019.” Publications Office of the European Union. <https://ec.europa.eu/regional_policy/sources/docgener/work/2019_03_rci2019.pdf>.

Gelman, A. and J. Hill. 2006. *Data Analysis Using Regression and Multilevel/hierarchical Models*. New York: Cambridge University Press.

Oesch, D. 2013. *Occupational Change in Europe. How Technology & Education Transform the Job Structure*. Oxford: Oxford University Press.

1. We chose to avoid a more complex cross-classified structure for several reasons. First, our data are not panel but repeated cross-section, hence the observations within the same region and country but observed in different years represent by all means different populations. Using region/year and country/year units therefore reflects better the stochastic process generating the data and takes also into account the time-variant nature of our predictor of labour market structure. Second, our main predictor qualifying the labour market at the regional level has a different value for every year, to take into account the change of labour markets (Oesch, 2013; Author, 2019). Third and final, this strategy allows us to maximise the number of level-2 units, improving the power of our analysis. [↑](#footnote-ref-1)
